# Cancer therapy‐related cardiotoxicity is associated with distinct alterations of the myocardial lipidome

**DOI:** 10.1002/ejhf.3656

**Published:** 2025-05-01

**Authors:** Vera M. Braun, Anna Foryst‐Ludwig, Ulf Landmesser, István Baczkó, Hendrik Milting, Ulrich Kintscher, Niklas Beyhoff

**Affiliations:** ^1^ Institute of Pharmacology Max Rubner Center for Cardiovascular Metabolic Renal Research, Charité ‐ Universitätsmedizin Berlin Berlin Germany; ^2^ DZHK (German Centre for Cardiovascular Research), Partner Site Berlin Berlin Germany; ^3^ Department of Cardiology, Angiology and Intensive Care Medicine Deutsches Herzzentrum der Charité Berlin Germany; ^4^ Department of Pharmacology and Pharmacotherapy, Albert Szent‐Györgyi Medical School University of Szeged Szeged Hungary; ^5^ Erich and Hanna Klessmann Institute for Cardiovascular Research and Development, Clinic for Thoracic and Cardiovascular Surgery, Heart and Diabetes Center NRW Bad Oeynhausen Germany

**Keywords:** Anthracyclines, Lipidomics, Lipids, Cardiomyopathy, Cancer therapy‐related cardiac dysfunction, Cardiotoxicity

## Abstract

**Aims:**

Anthracyclines are key components of various chemotherapy regimens, but their clinical utility is limited by severe cardiotoxic side effects. Previous studies have suggested that anthracycline‐induced cardiotoxicity (AIC) may be driven by alterations in myocardial lipid metabolism. This study aimed to systematically explore the cardiac lipidomic landscape of AIC with regard to potential pathomechanisms and novel therapeutic targets.

**Methods and results:**

Mass spectrometry‐based untargeted lipidomics were performed on myocardial biopsies from 13 patients with AIC (age 53 ± 31 years, 54% female, left ventricular ejection fraction 19 ± 4%) and 15 age‐ and sex‐matched controls. Lipidomic profiles were also compared with 15 patients with other heart failure aetiologies (matched for sex and age). A total of 627 individual lipid species from 22 different lipid classes were analysed. AIC was associated with a lower proportion of polyunsaturated fatty acids towards more monounsaturated and saturated sidechains as well as significant alterations in the proportion of odd‐chain fatty acids. Pathway analyses indicated a higher precursor conversion into lysolipids in AIC. This accumulation of lysolipid species was not observed in other heart failure aetiologies and may represent a specific finding in AIC.

**Conclusion:**

Anthracycline‐induced cardiotoxicity leads to distinct alterations of the myocardial lipidome. Increased levels of myocardial lysolipids were identified as a novel lipidomic trait in AIC, which appears to be distinct from other causes of heart failure. Further research studying pharmacological interventions in lysolipid metabolism for prevention and therapy of AIC is warranted.

## Introduction

Anthracyclines are highly effective anticancer drugs and represent key components of various contemporary chemotherapy regimens. Their clinical utility, however, is limited by dose‐dependent cardiotoxic side effects causing substantial long‐term complications in cancer survivors.^1^, ^2^, ^3^ A well‐established manifestation of anthracycline‐induced cardiotoxicity (AIC) is left ventricular systolic dysfunction that frequently progresses to heart failure development. Recent registry data indicated that the prevalence of heart failure is as high as 5% in contemporary cancer survivors previously treated with anthracyclines.^4^ Although various mechanisms underlying AIC have been proposed, specific treatment strategies to prevent these deleterious effects are currently lacking.

A growing body of evidence suggests that lipids may play a central role in the complex and multifactorial pathophysiology of AIC. As a key pathogenetic factor, anthracyclines induce the generation of reactive oxygen species that cause mitochondrial damage and dysfunction via peroxidation of membrane lipids.^3^, ^5^, ^6^ In addition to oxidative damage, anthracyclines have been shown to build irreversible complexes with lipids of the inner mitochondrial membrane that interfere with the electron transport chain.^7^, ^8^, ^9^, ^10^, ^11^ AIC is associated with profound derangements of myocardial metabolism including utilisation of fatty acids. Furthermore, pharmacological interventions affecting lipid metabolism have been shown to mitigate AIC in both pre‐clinical and patient studies.^12^, ^13^, ^14^ However, systematic investigations of the impact of AIC on the myocardial lipid composition in patients are currently missing.

In the present study, we aimed to characterise the myocardial lipidomic landscape in patients with AIC. By using large‐scale mass spectrometry‐based lipidomics of myocardial biopsies, we provide a comprehensive map of the global distribution and concentrations of lipid classes and species in heart failure following AIC and compare those with other heart failure aetiologies (oHF) and non‐failing controls.

## Methods

The study was approved by the institutional ethics committee (EA4/124/21, Ethics Committee of Charité ‐ Universitätsmedizin Berlin, Germany) and complied with the Declaration of Helsinki.

### Myocardial tissue acquisition

Human left ventricular biopsies were collected for research purposes under approval from local ethics committees (21/2013, Ruhr‐University Bochum, located in Bad Oeynhausen, Germany; 4991–0/2010‐1018EKU [339/PI/010], Scientific and Research Ethical Committee of the Medical Scientific Board at the Hungarian Ministry of Health [ETT‐TUKEB], Hungary). Non‐failing myocardium was obtained from organ donors with no previous history of cardiovascular disease whose hearts could not have been used for transplantation due to logistical reasons. Samples from patients with AIC and oHF were collected during heart transplantation (*n* = 16) or implantation of a left ventricular assist device (*n* = 12). Patients in the oHF group presented with ischaemic cardiomyopathy (*n* = 8) and (non‐ischaemic) dilated cardiomyopathy (*n* = 7).

All participants in the AIC and oHF group provided written informed consent; samples from organ donors were procured under national presumed consent legislation in accordance with ETT‐TUKEB regulations. Unfixed myocardial samples were snap‐frozen and stored in liquid nitrogen or at −80°C until further processing.

### Lipidomic analysis

For lipidomics, frozen tissue samples were pulverised and suspended in distilled water (final concentration 5 mg/ml). Mass spectrometry‐based lipidomics was conducted by Lipotype GmbH (Dresden, Germany), as previously described.^15^ Lipid extraction was executed using a two‐step chloroform/methanol procedure.^16^ Internal standard mixtures were added to the samples, these included species from different classes: cardiolipins, ceramides, diacylglycerols, hexosyl‐ceramides, lysophosphatidic acid, lysophosphatidylethanolamine, lysophosphatidylglycerol, lysophosphatidylinositol, lysophosphatidylserine, phosphatidic acid, phosphatidyl‐choline, phosphatidylethanolamine, phosphatidylglycerol, phosphatidylinositol, phosphatidylserine, cholesterol ester, sphingomyelin, and triacylglycerols. The organic phase was transferred to an infusion plate and dried using a speed vacuum concentrator after extraction. Dry extract from the first step was reconstituted in 7.5 mM ammonium acetate in chloroform/methanol/propanol (1:2:4; V:V:V), while dry extract from the second step was resuspended in a 33% ethanol solution of methylamine in chloroform/methanol (0.003:5:1; V:V:V). Liquids were handled with the Hamilton Robotics STARlet robotic platform equipped with the anti‐droplet control feature for precise pipetting of organic solvents.

Analysis was carried out by direct infusion of samples on a QExactive mass spectrometer (Thermo Scientific) using a TriVersa NanoMate ion source (Advion Biosciences). The analysis was performed in both positive and negative ion modes, with a resolution of Rm/z = 200 = 280 000 for mass spectrometry and Rm/z = 200 = 17 500 for tandem mass spectrometry experiments, within a single acquisition. Tandem mass spectrometry was triggered using an inclusion list including relevant mass spectrometry mass ranges scanned in 1 Da steps.^17^ Mass spectrometry and tandem mass spectrometry data were joined to track cholesterol ester‐, diacylglycerol‐ and triacylglycerol‐ions as ammonium adducts; phosphatidylcholine, phosphatidylcholine‐ether as acetate adducts; and cardiolipin, phosphatidic acid, phosphatidylethanolamine, phosphatidylethanolamine‐ether, phosphatidylglycerol, phosphatidylinositol and phosphatidylserine as deprotonated anions. Mass spectrometry alone was applied to track lysophosphatidic acid, lysophosphatidylethanolamine, lysophosphatidylethanolamine‐ether, lysophosphatidylglycerol, lysophosphatidylinositol and lysophosphatidylserine as deprotonated anions; ceramide, hexosylceramide, lysophosphatidylcholine, lysophosphatidylcholine‐ether and sphingomyelin as acetate adducts.

Data were analysed using an in‐house LipidXplorer‐based lipid identification software,^18^, ^19^ post‐processing and normalization were conducted with an in‐house data management system. Solely lipid identifications with a signal‐to‐noise ration greater than 5 and signal intensity at least 5 times higher than the corresponding blank sampled were considered for further analysis.

### Clinical data collection

Demographics, clinical information, and echocardiography data were extracted from patients' medical records. All data were acquired during clinical routine. AIC was defined as heart failure following anthracycline‐based chemotherapy in the absence of an alternative disease cause, corresponding to ‘very severe cancer therapy‐related cardiac dysfunction’ as defined by the European Society of Cardiology guidelines on cardio‐oncology.^20^

### Statistics

Statistical analysis was carried out using R version 4.3.2. Pmole data of lipid species was normalized to the total pmole of all measured lipids in each sample. For analyses on the lipid species level, data were filtered for at least three valid measurements per lipid species in each cohort. For methods that cannot deal with missing data, median imputation was applied. Normality was tested using the Shapiro–Wilk test. For normally distributed data the Welch t‐test was applied, while Mann–Whitney U test and Kruskal–Wallis test (for multiple groups) were used for data that were not normally distributed. Categorical data were tested using the chi‐squared test. *P*‐values were adjusted for multiple testing using the false discovery rate (FDR) method of Benjamini and Hochberg. The following levels of significance were defined: ****< 0.0001, ***< 0.001, **< 0.01, *< 0.05, ns > 0.05. Differential alterations were defined as |log2(FC)| >1, effect size was calculated using Cliff's delta statistic. Fisher's exact test was applied to test for enrichment. Lipid MAPS' BioPAN was used to analyse reactions,^21^ data normalized to total lipid content were uploaded to the software and the *p*‐value cut‐off was set to 0.05.

## Results

### Study population

Left ventricular biopsies from a total of 43 age‐, sex‐, and body mass index‐matched individuals were analysed of which 13 had AIC, 15 had oHF, and 15 were non‐failing controls. Baseline characteristics and clinical features of the study cohort are displayed in *Table* *1*. Patients with AIC and oHF were compared to a non‐failing control group comprising of organ donors without cardiac conditions (left ventricular ejection fraction [LVEF] 65 ± 5%) whose hearts could not have been used for transplantation. All heart failure patients were New York Heart Association functional class III or IV despite guideline‐directed medical therapy. Aetiologies in the oHF group included ischaemic (53%) and dilated cardiomyopathy (47%). Body mass index was comparable among groups with a slightly lower proportion of overweight/obese individuals in AIC. While LVEF was lower in AIC than in oHF (19 ± 4 vs. 25 ± 5%, *p* < 0.001), left ventricular dilatation was less pronounced (left ventricular end‐diastolic diameter: 62 ± 6 mm vs. 69 ± 7 mm, *p* < 0.05).

**Table 1:** Baseline characteristics of the study cohort

	Control	AIC	oHF	*p*‐value
*n*	15	13	15	
Demographics				
Female sex, %	53.3 (8/15)	53.8 (7/13)	53.3 (8/15)	1.00^a^
Age, years	56 ± 6	53 ± 13	50 ± 12	0.11^a^
BMI, kg/m^2^	26.7 ± 4.6	24.0 ± 4.4	26.9 ± 2.4	0.11^a^
Overweight/obese (BMI ≥25 kg/m^2^), %	50 (7/14)	30.8 (4 /13)	71.4 (10/14)	0.11^a^
Diabetes, %	0 (0/15)	23.1 (3/13)	28.6 (4/14)	0.16^a^
aHT, %	40.0 (6/15)	38.5 (5/13)	21.4 (3/14)	0.61^a^
Dyslipidaemia, %	0 (0/15)	38.5 (5/13)	42.9 (6/14)	**0.060** ^a^
Atrial fibrillation, %	0 (0/15)	23.1 (3/13)	50.0 (7/14)	**0.025** ^a^
Clinical characteristics				
NYHA class III, %	–	15.4 (2/13)	60.0 (6/10)	**0.032** ^b^
NYHA class IV, %	–	84.6 (11/13)	40.0 (4/10)	
LVEF, %	65 ± 5	19 ± 4	25 ± 5	**0.004** ^b^
LVEDd, mm	–	62 ± 6	69 ± 7	**0.031** ^b^
IVSd, mm	–	8 ± 2	10 ± 3	0.22^b^
LA diameter, mm	–	47 ± 6	51 ± 8	0.18^b^
SBP, mmHg	123 ± 12	102 ± 14	105 ± 14	**<0.001** ^a^
DBP, mmHg	71 ± 10	66 ± 13	70 ± 14	0.67^a^
Medication				
Beta‐blocker, %	13.3 (2/15)	84.6 (11/13)	100 (13/13)	0.91^b^
ACEi, %	20.0 (3/15)	46.2 (6/13)	30.8 (4/13)	0.86^b^
ARB, %	0 (0/15)	30.8 (4/13)	46.2 (6/13)	0.86^b^
MRA, %	0 (0/15)	61.5 (8/13)	76.9 (10/13)	0.90^b^
Diuretic, %	0 (0/15)	100 (13/13)	92.3 (12/13)	0.96^b^
SGLT2i, %	0 (0/15)	7.7 (1/13)	0 (0/13)	0.78^b^
Statin, %	0 (0/15)	15.4 (2/13)	53.8 (7/13)	0.64^b^
Insulin, %	0 (0/15)	15.4 (2/13)	23.1 (3/13)	0.90^b^

ACEi, angiotensin‐converting enzyme inhibitor; aHT, arterial hypertension; AIC, anthracycline‐induced cardiotoxicity; ARB, angiotensin receptor blocker; BMI, body mass index; DBP, diastolic blood pressure; IVSd, end‐diastolic interventricular septum thickness; LA, left atrial; LVEDd, left ventricular end‐diastolic diameter; LVEF, left ventricular ejection fraction; MRA, mineralocorticoid receptor antagonist; NYHA, New York Heart Association; oHF, other heart failure aetiology; SBP, systolic blood pressure; SGLT2i, sodium–glucose cotransporter 2 inhibitor.

Variables are displayed as frequency or mean ± SD as appropriate. For comparison of categorical data, chi‐squared test was used; for multiple comparisons one‐way ANOVA (normally distributed data) or Kruskal–Wallis test (for not normally distributed data) and Student's *t*‐test for single comparisons (of normally distributed data) were used.

^a^ Comparison between control, AIC, and oHF.

^b^ Comparison between AIC and oHF.

Information on malignant disease and corresponding treatment in the AIC group are detailed in online supplementary *Tables* *S1* and *S2*. Most common cancer types were non‐Hodgkin's lymphoma, breast cancer, and sarcomas. The majority received anthracyclines together with other antineoplastic therapy, most commonly as a part of the CHOP regimen together with cyclophosphamide, vincristine, and prednisolone; 23.1% underwent additional chest radiotherapy. One individual in the AIC group was treated for a paediatric cancer. Time between first chemotherapy and sample collection ranged from 5 to 39 years (median 13 years). The median time from last chemotherapy until sample collection was 9 (2–37) years.

### Characterisation of the global myocardial lipidome in anthracycline‐induced cardiotoxicity

**Figure 1:**
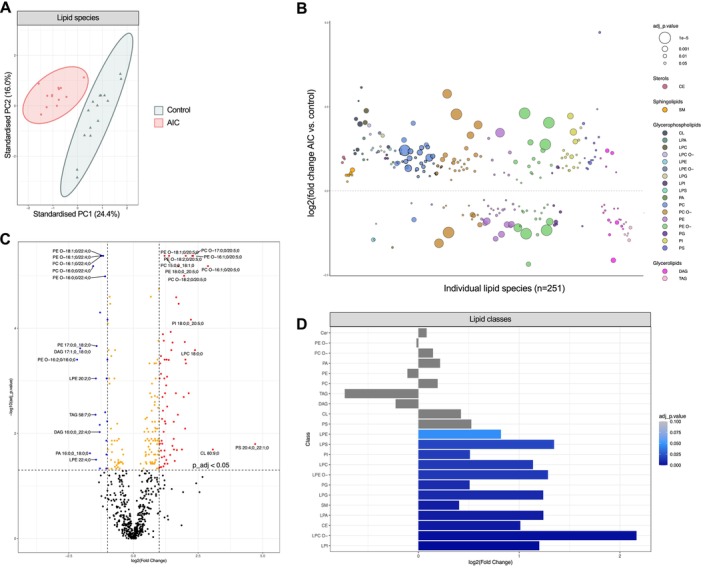
Characterisation of the global myocardial lipidome in AIC. (*A*) Principal component analysis (PCA) of filtered lipid species. (*B*) Bubble graph showing log2(fold change) of lipid species in AIC versus controls (filtered for adjusted *p*‐value < 0.1). Bubble size is inversely proportional to *p*‐value. Colour represents lipid class. Species above horizontal dashed line (at log2[fold change] = 0) are increased in AIC, below decreased. (*C*) Volcano plot of all lipid species. Blue shows significantly and differentially down‐regulated lipid species, red significantly and differentially up‐regulated species. Orange shows significantly but not differentially altered species. Horizontal dashed line at adjusted *p*‐value = 0.05, all species above the line are significantly altered. Vertical dashed lines at 1 and −1 log2(fold change), lipid species outside of these lines show differential divergence. (*D*) Bar graph showing the fold change of lipid classes in AIC versus control. Colour represents the adjusted *p*‐value, deeper blue depicting lower adjusted *p*‐values. CE, cholesterol ester; Cer, ceramide; Chol, cholesterol; CL, cardiolipin; DAG, diacylglycerol; GL, glycerolipid; GPL, glycerophospholipid; LPA, lysophosphatidate; LPC O‐, lysophosphatidylcholine (‐ether); LPE O‐, lysophosphatidylethanolamine (‐ether); LPI, lysophosphatidylinositol; LPS, lysophosphatidylserine; PA, phosphatidate; PC O‐, phosphatidylcholine (‐ether); PE O‐, phosphatidylethanolamine (‐ether); PG, phosphatidylglycerol; PI, phosphatidylinositol; PS, phosphatidylserine; SM, sphingomyelin; TAG, triacylglycerol.

First, the global lipidome was compared between controls and AIC to understand its impact on myocardial lipid distribution and concentration (*Figure* *1*). After filtering for at least three valid measurements per lipid species and study group, a total of 627 individual lipid species were identified spanning four different structural categories: glycerophospholipids (75.4% of number of lipid species measured), glycerolipids (20.4%), sphingolipids (3.5%), and sterol ester lipids (0.6%). The majority (83.3%) were membrane components, the others represented storage lipids (10.0%) or lysolipids (6.7%).

The absolute amount of lipids was comparable between controls and AIC patients (12.65 [10.23–15.07] vs. 12.68 [9.50–15.85] nmole, *p* = 0.70). Principal component analysis of lipid species separated the groups into two clusters with 40.4% of variance explained by the first two principal components (*Figure* *1A*). These results were most strongly determined by glycerophospholipids. A total of 91 lipid species were significantly and differentially regulated, representing 19 different lipid classes (out of 22 lipid classes analysed) (*Figure* *1B,C*).

The most abundant lipid class in both groups was phosphatidylcholine; triacylglycerols were the second most common lipid class in controls, whereas it was less abundant in AIC (data not shown). Twelve lipid classes were significantly upregulated in AIC: lysophosphatidylcholine‐ether, lysophosphatidylinositol, lysophosphatidic acid, lysophosphatidylglycerol, cholesterol ester, lysophosphatidylethanolamine‐ether, lysophosphatidylserine, lysophosphatidylcholine, phosphatidylinositol, sphingomyelin, and phosphatidylglycerol (*Figure* *1D*). While changes in cardiolipins failed to reach statistical significance at class level (*Figure* *1D*), cardiolipin 80:9;0 and cardiolipin 68:7;0 were among the top ten enriched lipid species in AIC (*Figure* *1C*).

### Lipid pathway analysis

**Figure 2:**
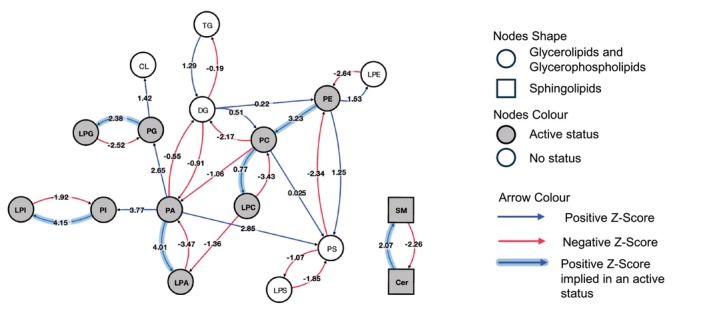
Most active reactions of lipid classes in AIC (generated with Lipid MAPS' BioPan).

We next mapped the complex network of lipid reactions and compared their activation status between patients with AIC and controls (*Figure* *2*, online supplementary *Table* *S3*). The conversion of phospholipids to lysophospholipids represented the most active reactions among lipid classes. At the single lipid species level, the most active reactions included the conversion of DG(17:1_18:0) to PC(17:1_18:0), PA(16:0_18:0) to PS(16:0_18:0), and PE(18:1_22:4) to PS(18:1_22:4). Other significantly activated reactions result in an increase of cardiolipin, phosphatidylserine and sphingomyelin. Lysolipid‐reactions using lysophosphatidylserine, lysophosphatidylethanolamine, lysophosphatidylglycerol, lysophosphatidylinositol, lysophosphatidic acid and lysophosphatidylcholine as substrates were most suppressed, as well as non‐lysolipid reactions using phosphatidylserine, phosphatidylcholine, and sphingomyelin as substrates. The Kennedy pathway, producing phosphatidic acid from lysophosphatidic acid, was amongst the most suppressed pathways.

### Fatty acid carbon chain length and double bonds

**Figure 3:**
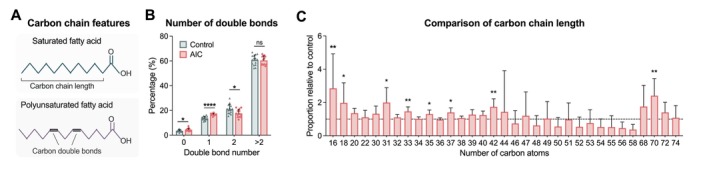
Characterisation of fatty acid carbon chain length and double bonds. (*A*) Schematic illustration of carbon chain length and number of carbon–carbon double bonds as chemical features of fatty acids. (*B*) Number of carbon double bonds in AIC and controls. (*C*) Comparison of fatty acid abundance according to carbon chain length. Displayed is the proportion of fatty acids in AIC patients relative to controls. *p < 0.05, **p < 0.01, ****p < 0.0001, using Wilcoxon rank test, adjusted for multiple testing using false discovery rate method of Benjamini and Hochberg (*B* and *C*).

Further structural analysis of the myocardial lipid composition revealed a lower proportion of polyunsaturated fatty acids (two or more double bonds) towards more monounsaturated and saturated sidechains in AIC (*Figure* *3A,B*). Compared to controls, patients with AIC showed an increased proportion of lipids with a chain length of less than 20 carbon atoms (*Figure* *3C*). Among fatty acids with a chain length between 30 and 40 carbon atoms, significant differences were observed in the proportion of odd‐chain fatty acids (consisting of 31, 33, 35, and 37 atoms, respectively) (*Figure* *3C*).

### Anthracycline‐induced cardiotoxicity is associated with an increase in lysolipids

**Figure 4:**
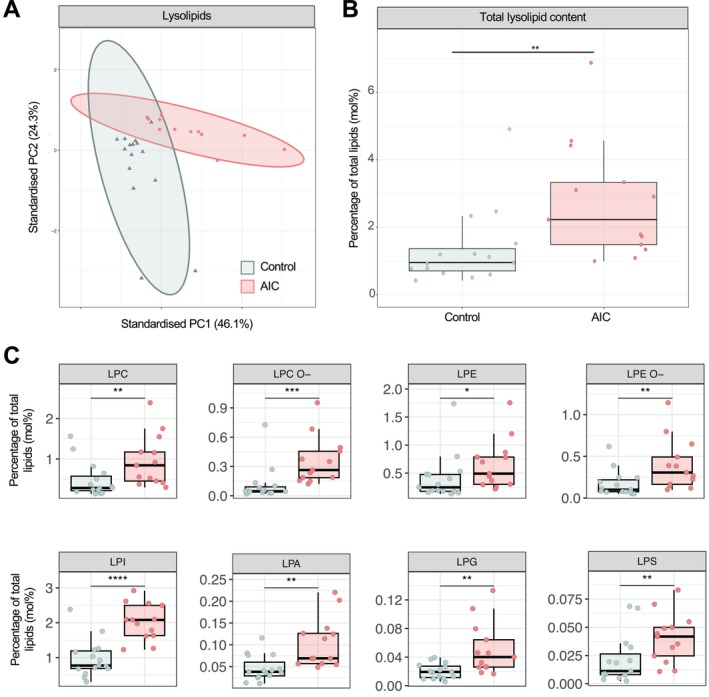
Lysolipids are significantly altered in AIC. (*A*) Principal component (PC) analysis of lysolipid species (filtered). (*B*) Boxplot showing mole‐percent lysolipids of total lipid content. There is a significant increase in lysolipids in AIC. (*C*) Relative proportion of the lysolipid classes AIC versus control.

Based on the observed increase in lysolipid conversion, we next performed a comparative analysis of the corresponding lipid species (*Figure* *4*). As expected, the overall abundance of lysophospholipids was increased in AIC, although principal component analysis of lysolipid species showed some clustering but a partial overlap with controls (*Figure* *4A,B*). The top three loading scores were represented by lysophosphatidylcholine16:0;0 (0.222), lysophosphatidylethanolamine‐ether18:2;0 (0.221), and lysophosphatidylcholine‐ether18:2;0 (0.220). All eight lysolipid classes were significantly increased in AIC compared to control and showed, except for lysophosphatidylethanolamine, a log2 fold‐change (FC) of greater than one.

### Alterations in lysolipids are specific to anthracycline‐induced cardiotoxicity

**Figure 5:**
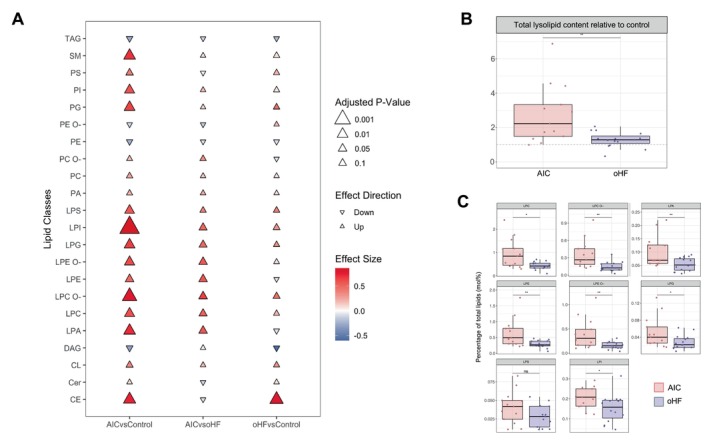
Specific lipid alterations in AIC compared to other heart failure aetiologies (oHF). (*A*) Cuneiform plot of the measured lipid classes in three comparisons: AIC versus control, AIC versus oHF, and oHF versus control. (*B*) Total lysolipid content, relative to the lysolipid content of the control group (= 1, dashed line). (*C*) Boxplot of lipid classes in AIC compared to oHF in mole% of total lipid content.

To evaluate if the observed lipidome changes represent specific features of AIC rather than general hallmarks of heart failure, myocardial lipidomics data from patients with oHF were integrated into the analyses (*Figure* *5*). The functional category of lysolipids was also significantly increased in AIC compared to oHF. Seven lysolipid classes were significantly increased: lysophosphatidylcholine, lysophosphatidylcholine‐ether, lysophosphatidylethanolamine, lysophosphatidylethanolamine‐ether, lysophosphatidic acid, lysophosphatidylglycerol, lysophosphatidylinositol. Lysophosphatidylcholine‐ether and lysophosphatidylethanolamine‐ether also showed a log2(FC) >1. After *p*‐value adjustment, no individual lipid species were significantly altered between AIC and oHF (online supplementary *Figure* *S1*).

## Discussion

This study of the largest human myocardial sample set of AIC investigated remodelling of the cardiac lipidome in patients with end‐stage disease (*Graphical Abstract*). While the focus of previous studies was mainly on specific lipids and certain pathways in lipid metabolism, systematic investigations of the entire myocardial lipidome have not yet been reported.

A computational study focusing on anthracycline–membrane interactions indicated that specific interactions between membranes and anthracycline molecules are crucial in the cardiotoxic effects of anthracyclines. Although the authors applied a sophisticated membrane model, a main limitation was the relatively low complexity of the model, including a lack of various membrane lipids.^11^ The lipidomic changes after anthracycline exposure observed in the present study may thus be used to inform and refine corresponding *in‐silico* models.

In previous studies on specific lipid interactions of anthracyclines, cardiolipin was a main focus.^7^, ^8^ MTP‐131, a peptide stabilising cardiolipin, was found effective in mitigating AIC in corresponding cell culture and mouse models.^22^, ^23^ In the present study of human end‐stage AIC, we observed an enrichment of single cardiolipin species, but no systematic change at overall class level. It appears likely that remodelling of individual lipid species depends on various factors including species (human vs. rodents), time course (years vs. weeks/months), and disease stage (end‐stage heart failure vs. early AIC), and future research is needed to gain a mechanistic understanding of these molecular changes. Interestingly, interactions between anthracyclines and cardiolipins seem to be similar to interactions with other anionic phospholipids such as phosphatidylserine, phosphatidylinositol and phosphatidic acid,^9^, ^24^ of which some species were significantly regulated in the present study.

We identified a lipidomic fingerprint in AIC that is characterised by significant alterations of more than 90 lipid species and 11 lipid classes. On all levels of analysis, the most striking changes were observed in the composition of lysolipids. Myocardial lysolipids were not only more abundant in AIC compared to their respective control, but also in comparison to oHF. This implies that the accumulation of lysolipids may be specific to AIC, which warrants further investigation into their role as potential disease drivers and treatment targets.

Given that molecular analysis of myocardial tissue requires invasive procedures such as endomyocardial biopsies, identification of circulating metabolites reflecting observed cardiac lipidomic changes could lead to novel diagnostic markers. In line with our results, a previous study demonstrated alterations in circulating levels of phospholipids and lysophospholipids in breast cancer patients treated with anthracyclines.^25^ Importantly, these metabolomic changes correlated with the degree of cardiac injury, highlighting a potential role as biomarkers.^25^

Lysolipids were long considered as mere intermediates of phospholipid metabolism, however, distinct roles of lysolipids have recently been identified. Lysolipid metabolism is complex involving different pathways, they can be synthesized from and to their parent phospholipid (Land's cycle involving phospholipases A2 and membrane bound O‐acyl transferases/lysophosphatidylcholine acyltransferase),^26^ or from other lysolipids (e.g. autotaxin turns various lysolipids into lysophosphatidic acid).^27^ They are structurally important for membrane remodelling and rigidity and are involved in signalling pathways via specific receptors.^28^ Moreover, oxidized lysophospholipids are bioactive molecules promoting inflammation, which is believed to play a central role in several cardiovascular diseases including AIC.^29^, ^30^

Lysophosphatidic acid is the most abundant circulating lysolipid. To date, six specific G protein‐coupled receptors to lysophosphatidic acid have been identified. They have been shown to play a role in cell proliferation and migration, immune regulation, vascular homeostasis, and fibrosis.^31^ Lysophosphatidic acid is associated with adverse effects on cardiac function in post‐myocardial infarction animal models, as well as hypertrophy in a cell model through inhibition of autophagy.^32^ Circulating lysophosphatidic acid plays a role in inflammation and fibrosis in various organs. Ablation of one of its receptors (lysophosphatidic acid receptor 1) exerts cardioprotective effects against hypertrophic cardiomyopathy in mice.^25^

Induction of myocardial infarction in rats leads to an accumulation of mitochondrial lysophosphatidylethanolamine and lysophosphatidylcholine, which is accompanied by a decrease of the respective phosphatidylethanolamine and phosphatidylcholine, indicating an increased degradation of phospholipids to lysophospholipids through phospholipases.^34^ Interestingly inhibition of the mitochondrial calcium‐independent phospholipase A2 seems to be cardioprotective by diminishing this conversion in experimental models.^35^ On the other hand, anthracyclines have shown to inhibit this enzyme in a cell model.^36^

Lysophosphatidylserines have specific receptors and play a role in inflammation, cytokinesis, calcium signalling, and cell proliferation. Alterations in their metabolism and signalling are involved in the pathogenesis of different neurological and autoimmune diseases.^37^ Furthermore, lysophosphatidylserine was linked to myocardial necrotic cell death in a mouse model of cardiac pressure overload through mediation of G protein‐coupled receptor 34.^38^

In face of this collective evidence, the observed accumulation of lysolipids in the heart of patients with AIC may represent a new disease mechanism that warrants further investigation. Future studies should evaluate the potential of circulating biomarkers reflecting changes in the cardiac lipidome for diagnostic and prognostic purposes in AIC. Ultimately, pharmacological strategies targeting impaired lysolipid metabolism may represent a novel approach to mitigate cardiotoxicity. Lysophosphatidic acid receptor 1 antagonists were recently tested in clinical phase II trials for the treatment of idiopathic pulmonary fibrosis^39^ and diffuse cutaneous systemic sclerosis,^40^ and their application in the context of AIC warrants further consideration.

### Limitations

Although this is, to our knowledge, the largest cohort of human myocardial AIC samples analysed so far, the sample size of 13 specimens is a main limitation of the present study. As for any biopsy study, acquired samples may not adequately reflect intra‐organ heterogeneity (i.e. sampling error), and regional differences in myocardial lipid composition cannot be excluded. Biopsies were taken after diagnosis of AIC only, which is why temporal changes in myocardial lipid composition and the role of the baseline lipidome in AIC development need to be addressed in future prospective studies. Patients were referred to our heart centre (Heart and Diabetes Center NRW, Bad Oeynhausen, Germany) to undergo heart failure surgery, often after many years of oncological and cardiological care at other hospitals. Consequently, the presented data were based on patients' medical records resulting in limited availability of some clinical information (e.g. details on cancer treatment and time course of AIC). As the cumulative anthracycline dose was not consistently available, it remains unclear if lipidomic changes in AIC are dose‐dependent. This study is descriptive of long‐term changes seen in AIC and should be considered hypothesis‐generating for further investigations into the role of lysolipid metabolism in the pathogenesis, diagnosis, and prevention/treatment of AIC.

## Conclusions

Heart failure following AIC is associated with distinct alterations of the myocardial lipid composition. Lipidomic features of AIC comprise a lower proportion of polyunsaturated fatty acids towards more monounsaturated and saturated sidechains as well as significant alterations in the proportion of odd‐chain fatty acids. Accumulation of lysolipids may represent a specific feature of AIC, which is absent in oHF. Future studies on the role of these lipidomic changes in the pathogenesis and potential mitigation of AIC are warranted.

## Supporting information


**Appendix S1.** Supporting Information.
