# Supplementary material for: Cancer therapy‐related cardiotoxicity is associated with distinct alterations of the myocardial lipidome
Source: Eur J Heart Fail. 2025 May 1;27(6):1056–66. doi: 10.1002/ejhf.3656 (PMC12260325; doi:10.1002/ejhf.3656)
Supplement: Supplementary file 1 — Appendix S1. Supporting Information. [file EJHF-27-1056-s001.pdf]

Appendices

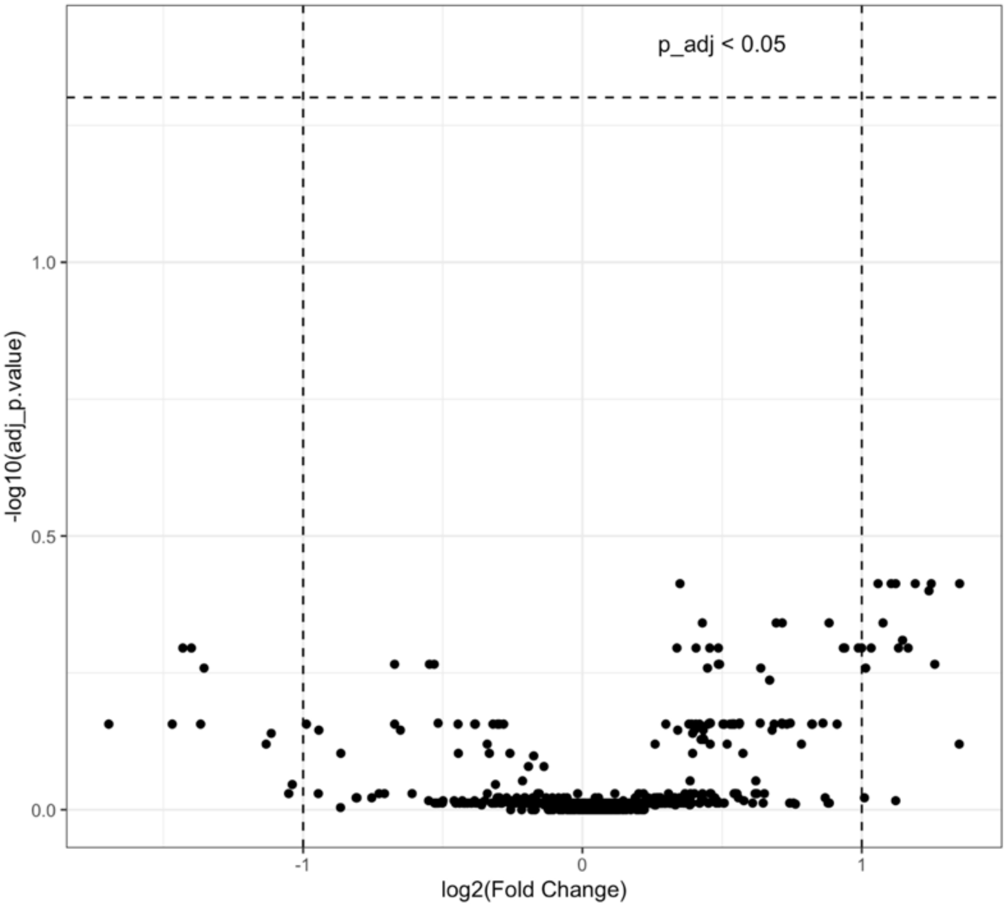

Supplemental Figure 1: Volcano plot of individual lipid species in AIC vs. oHF.

**Cancer types**	
Non-Hodgkin's lymphoma, %	53.8 (7/13)
Breast cancer, %	15.4 (2/13)
Sarcoma (Ewing sarcoma, Osteosarcoma), %	15.4 (2/13)
Acute myeloid leukemia, %	7.7 (1/13)
Wilms tumor, %	7.7 (1/13)
**Chemotherapy regimen**	
CHOP + Rituximab, %	30.8 (4/13)
CHOP + Etoposid, %	7.7 (1/13)
Epirubicin + Cyclophosphamide + 5-FU, %	7.7 (1/13)
Doxorubicin + Paclitaxel + Cyclophosphamid, %	7.7 (1/13)
Doxorubicin + Actinomycin D + Vincristin + Cyclophosphamide, %	7.7 (1/13)
Anthracycline (no further information available), %	38.5 (5/13)
**Thoracic radiotherapy, %**	23.1 (3/13)
**Median time since first chemotherapy, years [range]**	13 [5-39]
**Median time since last chemotherapy, years [range]**	9 [2-37]

Supplemental Table 1: Overview about cancer-related information of AIC group.

CHOP, cyclophosphamide + doxorubicin hydrochloride (hydroxydaunorubicin) + vincristine sulfate (Oncovin) + prednisone; 5-FU, 5-fluorouracil.

ID	♀	Age	Cancer	Chemotherapy	Years since first chemotherapy	Thoracic radiation	LVEF (%)	NYHA class	Cardiac surgery	BMI (kg/m^2)	DM	aHT	BNP (pg/ml)	NT-proBNP (pg/ml)
1	0	58	NHL (B-cell lymphoma)	CHOP + Etoposid	9		15	III	LVAD	28.1	y	y	3,110	
2	1	15	Wilms tumor	Anthracycline			20	IV	LVAD	23.1	n	n	2,870	
3	1	61	Breast cancer	Epirubicin + Cyclophosphamide + 5-FU	10	y	14	III	LVAD	23.6	n	n		
4	0	51	NHL (B-cell lymphoma)	Anthracycline			20	IV	LVAD	22.9	y	y	2,680	
5	0	66	NHL (B-cell lymphoma)	CHOP + Rituximab	8		12	IV	LVAD	31.3	n	y		7,640
6	0	52	Osteosarcoma	Doxorubicin	15		25	IV	HTx	28.4	y	y		3,050
7	1	56	Breast cancer	Doxorubicin + Paclitaxel + Cyclophosphamid	9		20	IV	LVAD	20.6	n	n		1,568
8	0	63	NHL	CHOP + Rituximab	13		15	IV	LVAD	21.3	n	y	4,120	
9	1	46	Ewing sarcoma	Doxorubicin + Actinomycin D + Vincristin + Cyclophosphamide	39	y	24	IV	LVAD	20.2	n	n		
10	1	58	Acute myeloid leukemia	Doxorubicin	16		18	IV	BVAD	19.0	n	n		
11	0	65	NHL (B-cell lymphoma)	CHOP + Rituximab	15	y	16	IV	LVAD	24.3	n	n		40,500
12	1	54	NHL	CHOP + Rituximab	5		20	IV	LVAD	30.8	n	n		
13	1	43	NHL	Anthracycline	21		25	IV	LVAD	17.9	n	n	2,420	13,100

Supplemental Table 2: Detailed characteristics of AIC patients.

aHT, arterial hypertension; BVAD, biventricular assist device; BMI, body mass index; BNP, B-type natriuretic peptide; CHOP, cyclophosphamide + doxorubicin hydrochloride (hydroxydaunorubicin) + vincristine sulfate (Oncovin) + prednisone; DM, diabetes mellitus; HTx, heart transplantation; LVAD, left ventricular sssist device; NHL, Non-Hodgkin lymphoma; 5-FU, 5-fluorouracil.

lipid class active reaction (AIC vs. Control)		
**Reactions chains**	Z-score	Predicted genes
**PA->PI->LPI**	5.601	CDS1, CDS2, CDIPT, PLA2G4D, PLA2G4E
**PI->LPI**	4.151	PLA2G4D, PLA2G4E
**PA->LPA**	4.008	PLA2G4A, PLA2G4D, PLA2G4E, PLA2G4F
**PA->PG->LPG**	3.55	CDS1, CDS2, PTPMT1, PLA2G4D, PLA2G4F, CRLS1
**PA->PG->CL**	2.874	CDS1, CDS2, PTPMT1, CRLS1
**PA->PS**	2.853	CDS1, PTDSS1
**PE->PC->LPC**	2.83	PEMT, PLA2G2E, PLA2G2A, PLA2G2D, PLA2G2F, PLA2G1B, PLA2G4A, PLA2G4B, PLA2G4C, PLA2G4D, PLA2G4E, PLA2G4F
**PG->LPG**	2.376	PLA2G4D, PLA2G4F, CRLS1
**PE->PC->PS**	2.302	PEMT, PTDSS1
**Cer->SM**	2.068	SGMS1, SGMS2, CERT1
**lipid class most active reaction (AIC vs. Control)**		
**Reactions chains**	Z-score	Predicted genes
**PI->LPI**	4.151	PLA2G4D, PLA2G4E
**PA->LPA**	4.008	PLA2G4A, PLA2G4D, PLA2G4E, PLA2G4F
**PE->PC->LPC**	2.83	PEMT, PLA2G2E, PLA2G2A, PLA2G2D, PLA2G2F, PLA2G1B, PLA2G4A, PLA2G4B, PLA2G4C, PLA2G4D, PLA2G4E, PLA2G4F
**PG->LPG**	2.376	PLA2G4D, PLA2G4F, CRLS1
**Cer->SM**	2.068	SGMS1, SGMS2, CERT1
**lipid class suppressed reaction (AIC vs. Control)**		
**Reactions chains**	Z-score	Predicted genes
**LPC->PC->DG->TG**	-3.342	LPCAT1, LPCAT2, LPCAT3, LPCAT4, DGAT2
**LPC->PC->PA**	-3.177	LPCAT1, LPCAT2, LPCAT3, LPCAT4, PLD1, PLD2
**LPC->PC->DG->PE**	-3.108	LPCAT1, LPCAT2, LPCAT3, LPCAT4, CEPT1
**LPS->PS->PE**	-2.958	MBOAT1, LPCAT3, LPCAT4, PISD
**LPE->PE**	-2.637	MBOAT1, MBOAT2, LPCAT3, LPCAT4
**LPG->PG**	-2.516	LPGAT1, CRLS1

**LPA->PA->DG->TG**	-2.427	AGPAT1, AGPAT2, AGPAT3, AGPAT4, AGPAT5, PLPP1, PLPP2, PLPP3, DGAT2
**LPC->PC->PS**	-2.408	LPCAT1, LPCAT2, LPCAT3, LPCAT4, PTDSS1
**PS->PE**	-2.335	PISD
**SM->Cer**	-2.264	SMPD1, SMPD4
**LPA->PA->DG->PE**	-2.192	AGPAT1, AGPAT2, AGPAT3, AGPAT4, AGPAT5, PLPP1, PLPP2, PLPP3, CEPT1
**PC->DG->PA**	-2.179	DGKA, DGKB, DGKD, DGKE, DGKG, DGKH, DGKI, DGKK, DGKQ, DGKZ
**LPC->PC->DG->PA->PS->LPS**	-2.116	LPCAT1, LPCAT2, LPCAT3, LPCAT4, DGKA, DGKB, DGKD, DGKE, DGKG, DGKH, DGKI, DGKK, DGKQ, DGKZ, CDS1, PTDSS1, PLA2G2E, PLA2G2A, PLA2G2F
**LPA->PA->DG->PC->PS->LPS**	-2.035	AGPAT1, AGPAT2, AGPAT3, AGPAT4, AGPAT5, PLPP1, PLPP2, PLPP3, CHPT1, PTDSS1, PLA2G2E, PLA2G2A, PLA2G2F
**LPC->PC->DG->PA->PG**	-1.934	LPCAT1, LPCAT2, LPCAT3, LPCAT4, DGKA, DGKB, DGKD, DGKE, DGKG, DGKH, DGKI, DGKK, DGKQ, DGKZ, CDS1, CDS2, PTPMT1
**LPI->PI**	-1.918	MBOAT7
**LPC->PC->DG->PA->PS->PE->LPE**	-1.823	LPCAT1, LPCAT2, LPCAT3, LPCAT4, DGKA, DGKB, DGKD, DGKE, DGKG, DGKH, DGKI, DGKK, DGKQ, DGKZ, CDS1, PTDSS1, PISD, PLA2G4C
**PC->DG->TG**	-1.667	DGAT2
**lipid class most suppressed reaction (AIC vs. Control)**		
**Reactions chains**	Z-score	Predicted genes
**LPS->PS->PE**	-2.958	MBOAT1, LPCAT3, LPCAT4, PISD
**LPE->PE**	-2.637	MBOAT1, MBOAT2, LPCAT3, LPCAT4
**LPG->PG**	-2.516	LPGAT1, CRLS1
**LPA->PA->DG->TG**	-2.427	AGPAT1, AGPAT2, AGPAT3, AGPAT4, AGPAT5, PLPP1, PLPP2, PLPP3, DGAT2
**PS->PE**	-2.335	PISD
**SM->Cer**	-2.264	SMPD1, SMPD4
**PC->DG->PA**	-2.179	DGKA, DGKB, DGKD, DGKE, DGKG, DGKH, DGKI, DGKK, DGKQ, DGKZ
**LPC->PC->DG->PA->PG**	-1.934	LPCAT1, LPCAT2, LPCAT3, LPCAT4, DGKA, DGKB, DGKD, DGKE, DGKG, DGKH, DGKI, DGKK, DGKQ, DGKZ, CDS1,

		CDS2, PTPMT1
**LPI->PI**	-1.918	MBOAT7
**lipid species active reaction (AIC vs. Control)**		
**Reactions chains**	Z-score	Predicted genes
**DG(17:1_18:0)->PC(17:1_18:0)**	6.213	CHPT1
**PA(16:0_18:0)->PS(16:0_18:0)**	5.532	CDS1, PTDSS1
**PE(18:1_22:4)->PS(18:1_22:4)**	5.362	PTDSS2
**PE(20:2_20:4)->PC(20:2_20:4)**	5.139	PEMT
**PA(38:5)->LPA(20:4)**	5.125	PLA2G4A, PLA2G4D, PLA2G4E, PLA2G4F
**PC(18:1_22:4)->PS(18:1_22:4)**	5.063	PTDSS1
**PE(O-17:2/18:1)->PC(O-17:2/18:1)**	4.659	PEMT
**PE(20:0_20:4)->PS(20:0_20:4)**	4.436	PTDSS2
**PE(O-18:2/18:1)->PC(O-18:2/18:1)**	4.42	PEMT
**DG(14:0_22:5)->PC(14:0_22:5)**	4.315	CHPT1
**PE(O-18:1/16:1)->PC(O-18:1/16:1)**	4.245	PEMT
**PC(16:0_20:1)->PS(16:0_20:1)**	4.245	PTDSS1
**DG(16:0_17:1)->PC(16:0_17:1)**	4.186	CHPT1
**PE(16:0_20:3)->PS(16:0_20:3)**	4.165	PTDSS2
**PE(16:0_18:2)->PS(16:0_18:2)**	4.123	PTDSS2
**PE(14:0_18:1)->PC(14:0_18:1)**	4.066	PEMT
**DG(17:1_18:1)->PC(17:1_18:1)**	4.034	CHPT1
**LPC(20:4)->LPA(20:4)**	4.031	ENPP2
**DG(15:0_18:1)->PC(15:0_18:1)**	4.012	CHPT1
**PE(16:1_22:4)->PS(16:1_22:4)**	3.999	PTDSS2
**PS(36:4)->LPS(20:4)**	3.989	PLA2G2E, PLA2G2A, PLA2G2F
**PE(16:0_22:6)->PC(16:0_22:6)**	3.93	PEMT
**PE(18:2_18:2)->PC(18:2_18:2)->PS(18:2_18:2)**	3.871	PEMT, PTDSS1
**PE(18:2_18:2)->PS(18:2_18:2)**	3.866	PTDSS2

**LPS(22:0)->PS(38:0)**	3.864	MBOAT1, LPCAT3, LPCAT4
**LPG(18:1)->PG(40:7)**	3.81	LPGAT1, CRLS1
**PI(36:4)->LPI(20:4)**	3.8	PLA2G4D, PLA2G4E
**PA(34:0)->LPA(16:0)**	3.756	PLA2G4A, PLA2G4D, PLA2G4E, PLA2G4F
**PA(16:0_18:0)->PG(16:0_18:0)**	3.712	CDS1, CDS2, PTPMT1
**PA(34:0)->LPA(18:0)**	3.696	PLA2G4A, PLA2G4D, PLA2G4E, PLA2G4F
**PE(18:1_20:1)->PS(18:1_20:1)**	3.629	PTDSS2
**PE(18:1_20:1)->PC(18:1_20:1)**	3.6	PEMT
**PA(18:0_18:2)->PI(18:0_18:2)**	3.576	CDS1, CDS2, CDIPT
**PE(18:1_18:2)->PS(18:1_18:2)**	3.462	PTDSS2
**PE(O-16:1/22:6)->PC(O-16:1/22:6)**	3.446	PEMT
**PE(16:0_20:2)->PC(16:0_20:2)**	3.427	PEMT
**PE(16:0_20:1)->PS(16:0_20:1)**	3.421	PTDSS2
**PE(O-16:1/16:1)->PC(O-16:1/16:1)**	3.41	PEMT
**PE(18:1_18:1)->PS(18:1_18:1)**	3.407	PTDSS2
**DG(16:1_20:3)->PC(16:1_20:3)**	3.316	CHPT1
**PE(16:1_18:1)->PC(16:1_18:1)->PA(16:1_18:1)->PI(16:1_18:1)**	3.299	PEMT, PLD1, PLD2, CDS1, CDS2, CDIPT
**PA(18:0_20:5)->PI(18:0_20:5)**	3.245	CDS1, CDS2, CDIPT
**PG(34:0)->LPG(18:0)**	3.229	PLA2G4D, PLA2G4F, CRLS1
**PE(17:0_20:4)->PC(17:0_20:4)**	3.221	PEMT
**PE(O-17:0/20:5)->PC(O-17:0/20:5)**	3.217	PEMT
**PE(18:1_22:5)->PC(18:1_22:5)**	3.214	PEMT
**PA(16:0_18:0)->DG(16:0_18:0)->PE(16:0_18:0)**	3.202	PLPP1, PLPP2, PLPP3, CEPT1
**Cer(36:2->#8594->O2)**	3.184	SGMS1, SGMS2, CERT1
**PE(16:0_16:0)->PC(16:0_16:0)**	3.16	PEMT
**PE(18:0_22:6)->PC(18:0_22:6)**	3.154	PEMT
**PA(18:1_18:2)->PS(18:1_18:2)**	3.152	CDS1, PTDSS1
**PC(18:1_18:2)->PS(18:1_18:2)**	3.145	PTDSS1

**PE(16:0_20:3)->PC(16:0_20:3)**	3.123	PEMT
**PE(18:1_20:3)->PS(18:1_20:3)**	3.118	PTDSS2
**PI(34:0)->LPI(18:0)->PI(40:6)**	3.076	PLA2G4D, PLA2G4E, MBOAT7
**PE(16:0_18:2)->PC(16:0_18:2)**	3.067	PEMT
**PA(18:1_20:4)->PG(18:1_20:4)**	3.052	CDS1, CDS2, PTPMT1
**PE(16:0_18:0)->PS(16:0_18:0)**	3.022	PTDSS2
**PG(36:4)->LPG(20:4)**	2.984	PLA2G4D, PLA2G4F, CRLS1
**PE(18:0_18:3)->PC(18:0_18:3)**	2.979	PEMT
**PE(O-16:1/16:0)->PC(O-16:1/16:0)**	2.964	PEMT
**DG(18:1_22:5)->PC(18:1_22:5)**	2.96	CHPT1
**PA(16:0_18:0)->PI(16:0_18:0)**	2.951	CDS1, CDS2, CDIPT
**Cer(42:2->#8594->O2)**	2.945	SGMS1, SGMS2, CERT1
**PC(16:0_18:2)->PS(16:0_18:2)**	2.934	PTDSS1
**PA(18:1_20:4)->PI(18:1_20:4)**	2.913	CDS1, CDS2, CDIPT
**PE(O-16:1/20:5)->PC(O-16:1/20:5)**	2.894	PEMT
**PC(18:1_20:3)->PS(18:1_20:3)**	2.858	PTDSS1
**LPC(16:1)->PC(32:2)**	2.83	LPCAT1, LPCAT2, LPCAT3, LPCAT4
**PE(O-17:0/17:1)->PC(O-17:0/17:1)**	2.829	PEMT
**PE(O-16:1/18:1)->PC(O-16:1/18:1)**	2.801	PEMT
**DG(18:1_20:1)->PC(18:1_20:1)->PS(18:1_20:1)**	2.799	CHPT1, PTDSS1
**PS(34:1)->LPS(18:1)**	2.697	PLA2G2E, PLA2G2A, PLA2G2F
**DG(16:0_22:6)->PC(16:0_22:6)**	2.689	CHPT1
**PA(16:0_18:2)->PS(16:0_18:2)**	2.644	CDS1, PTDSS1
**DG(18:1_22:6)->PE(18:1_22:6)**	2.619	CEPT1
**LPI(18:1)->PI(38:5)**	2.614	MBOAT7
**PC(34:1)->LPC(18:0)**	2.582	PLA2G2E, PLA2G2A, PLA2G2D, PLA2G2F, PLA2G1B, PLA2G4A, PLA2G4B, PLA2G4C, PLA2G4D, PLA2G4E, PLA2G4F
**PE(16:1_18:0)->PC(16:1_18:0)**	2.542	PEMT
**PE(18:2_20:1)->PS(18:2_20:1)**	2.513	PTDSS2

**PE(20:1_20:4)->PC(20:1_20:4)->PS(20:1_20:4)**	2.512	PEMT, PTDSS1
**PE(16:1_18:1)->PC(16:1_18:1)->DG(16:1_18:1)**	2.459	PEMT
**PC(32:0)->LPC(16:0)**	2.423	PLA2G2E, PLA2G2A, PLA2G2D, PLA2G2F, PLA2G1B, PLA2G4A, PLA2G4B, PLA2G4C, PLA2G4D, PLA2G4E, PLA2G4F
**PG(34:0)->LPG(16:0)**	2.416	PLA2G4D, PLA2G4F, CRLS1
**DG(18:0_22:6)->PC(18:0_22:6)**	2.416	CHPT1
**PE(O-18:1/22:4)->PC(O-18:1/22:4)**	2.413	PEMT
**Cer(34:2->#8594->O2)**	2.404	SGMS1, SGMS2, CERT1
**PE(34:1)->LPE(18:1)**	2.403	PLA2G4C
**PI(36:3)->LPI(20:3)**	2.392	PLA2G4D, PLA2G4E
**PE(18:1_18:1)->PC(18:1_18:1)->PA(18:1_18:1)**	2.391	PEMT, PLD1, PLD2
**PE(16:0_18:1)->PC(16:0_18:1)->PA(16:0_18:1)**	2.382	PEMT, PLD1, PLD2
**PE(O-16:0/20:5)->PC(O-16:0/20:5)**	2.356	PEMT
**PE(18:1_18:2)->PC(18:1_18:2)**	2.345	PEMT
**LPE(20:4)->PE(36:5)**	2.309	MBOAT1, MBOAT2, LPCAT3, LPCAT4
**PE(34:0)->LPE(18:0)**	2.287	PLA2G4C
**DG(16:1_20:4)->PE(16:1_20:4)**	2.279	CEPT1
**PE(20:1_20:4)->PS(20:1_20:4)**	2.276	PTDSS2
**PE(18:0_18:1)->PC(18:0_18:1)**	2.266	PEMT
**LPC(16:1)->PC(34:3)**	2.213	LPCAT1, LPCAT2, LPCAT3, LPCAT4
**PA(18:1_20:4)->PS(18:1_20:4)**	2.189	CDS1, PTDSS1
**PE(18:0_22:5)->PC(18:0_22:5)->DG(18:0_22:5)**	2.174	PEMT
**PE(O-18:0/20:5)->PC(O-18:0/20:5)**	2.174	PEMT
**PE(16:0_18:1)->PC(16:0_18:1)->PS(16:0_18:1)**	2.147	PEMT, PTDSS1
**PE(O-16:2/18:1)->PC(O-16:2/18:1)**	2.14	PEMT
**PE(18:1_18:3)->PC(18:1_18:3)-**	2.123	PEMT

**>DG(18:1_18:3)**		
**DG(18:0_20:2)->PC(18:0_20:2)**	2.12	CHPT1
**PG(34:2)->LPG(18:2)**	2.118	PLA2G4D, PLA2G4F, CRLS1
**PE(O-16:2/16:0)->PC(O-16:2/16:0)**	2.093	PEMT
**PA(16:1_18:0)->PG(16:1_18:0)**	2.062	CDS1, CDS2, PTPMT1
**DG(18:1_22:5)->PE(18:1_22:5)**	2.056	CEPT1
**PE(O-18:1/18:1)->PC(O-18:1/18:1)**	2.054	PEMT
**PE(32:0)->LPE(16:0)**	1.993	PLA2G4C
**PE(18:0_18:2)->PC(18:0_18:2)**	1.99	PEMT
**PA(16:1_18:1)->PI(16:1_18:1)**	1.987	CDS1, CDS2, CDIPT
**PE(18:0_20:2)->PS(18:0_20:2)**	1.97	PTDSS2
**PI(34:2)->LPI(18:2)->PI(34:3)**	1.963	PLA2G4D, PLA2G4E, MBOAT7
**PE(18:1_22:4)->PC(18:1_22:4)->DG(18:1_22:4)**	1.934	PEMT
**PC(18:1_20:1)->PS(18:1_20:1)**	1.916	PTDSS1
**DG(18:0_18:1)->PC(18:0_18:1)**	1.906	CHPT1
**PE(17:0_18:2)->PC(17:0_18:2)**	1.896	PEMT
**TG(50:1)->DG(34:1)**	1.893	PNPLA4, PNPLA5
**PE(O-16:1/22:5)->PC(O-16:1/22:5)**	1.882	PEMT
**PA(18:1_18:2)->PI(18:1_18:2)**	1.877	CDS1, CDS2, CDIPT
**PE(16:0_20:4)->PS(16:0_20:4)**	1.877	PTDSS2
**DG(18:1_22:6)->PC(18:1_22:6)**	1.875	CHPT1
**PE(18:0_22:4)->PC(18:0_22:4)->PS(18:0_22:4)**	1.864	PEMT, PTDSS1
**DG(18:2_22:5)->PC(18:2_22:5)**	1.833	CHPT1
**PE(O-17:1/18:2)->PC(O-17:1/18:2)**	1.825	PEMT
**PE(O-18:2/22:6)->PC(O-18:2/22:6)**	1.818	PEMT
**Cer(36:1->#8594->O2)**	1.818	SGMS1, SGMS2, CERT1
**PE(O-18:1/20:4)->PC(O-18:1/20:4)**	1.816	PEMT
**DG(16:0_18:1)->PC(16:0_18:1)**	1.788	CHPT1

**PE(18:0_20:2)->PC(18:0_20:2)**	1.783	PEMT
**PE(16:0_16:1)->PC(16:0_16:1)->DG(16:0_16:1)**	1.778	PEMT
**PE(O-16:0/18:2)->PC(O-16:0/18:2)**	1.749	PEMT
**PE(16:0_20:4)->PC(16:0_20:4)->PS(16:0_20:4)**	1.742	PEMT, PTDSS1
**TG(48:0)->DG(32:0)**	1.741	PNPLA4, PNPLA5
**PI(34:0)->LPI(18:0)->PI(40:5)**	1.693	PLA2G4D, PLA2G4E, MBOAT7
**PE(18:0_20:4)->PC(18:0_20:4)->DG(18:0_20:4)**	1.686	PEMT
**PE(18:0_22:5)->PC(18:0_22:5)->PS(18:0_22:5)**	1.666	PEMT, PTDSS1
**PA(16:1_18:0)->PS(16:1_18:0)->PE(16:1_18:0)->PC(16:1_18:0)->DG(16:1_18:0)**	1.655	CDS1, PTDSS1, PISD, PEMT
**lipid species most active reaction (AIC vs. Control)**		
**Reactions chains**	Z-score	Predicted genes
**DG(17:1_18:0)->PC(17:1_18:0)**	6.213	CHPT1
**PA(16:0_18:0)->PS(16:0_18:0)**	5.532	CDS1, PTDSS1
**PE(18:1_22:4)->PS(18:1_22:4)**	5.362	PTDSS2
**PE(20:2_20:4)->PC(20:2_20:4)**	5.139	PEMT
**PA(38:5)->LPA(20:4)**	5.125	PLA2G4A, PLA2G4D, PLA2G4E, PLA2G4F
**PC(18:1_22:4)->PS(18:1_22:4)**	5.063	PTDSS1
**PE(O-17:2/18:1)->PC(O-17:2/18:1)**	4.659	PEMT
**PE(20:0_20:4)->PS(20:0_20:4)**	4.436	PTDSS2
**PE(O-18:2/18:1)->PC(O-18:2/18:1)**	4.42	PEMT
**DG(14:0_22:5)->PC(14:0_22:5)**	4.315	CHPT1
**PE(O-18:1/16:1)->PC(O-18:1/16:1)**	4.245	PEMT
**PC(16:0_20:1)->PS(16:0_20:1)**	4.245	PTDSS1
**DG(16:0_17:1)->PC(16:0_17:1)**	4.186	CHPT1
**PE(16:0_20:3)->PS(16:0_20:3)**	4.165	PTDSS2
**PE(16:0_18:2)->PS(16:0_18:2)**	4.123	PTDSS2

**PE(14:0_18:1)->PC(14:0_18:1)**	4.066	PEMT
**DG(17:1_18:1)->PC(17:1_18:1)**	4.034	CHPT1
**LPC(20:4)->LPA(20:4)**	4.031	ENPP2
**DG(15:0_18:1)->PC(15:0_18:1)**	4.012	CHPT1
**PE(16:1_22:4)->PS(16:1_22:4)**	3.999	PTDSS2
**PS(36:4)->LPS(20:4)**	3.989	PLA2G2E, PLA2G2A, PLA2G2F
**PE(16:0_22:6)->PC(16:0_22:6)**	3.93	PEMT
**PE(18:2_18:2)->PC(18:2_18:2)->PS(18:2_18:2)**	3.871	PEMT, PTDSS1
**LPS(22:0)->PS(38:0)**	3.864	MBOAT1, LPCAT3, LPCAT4
**LPG(18:1)->PG(40:7)**	3.81	LPGAT1, CRLS1
**PI(36:4)->LPI(20:4)**	3.8	PLA2G4D, PLA2G4E
**PA(34:0)->LPA(16:0)**	3.756	PLA2G4A, PLA2G4D, PLA2G4E, PLA2G4F
**PE(18:1_20:1)->PS(18:1_20:1)**	3.629	PTDSS2
**PA(18:0_18:2)->PI(18:0_18:2)**	3.576	CDS1, CDS2, CDIPT
**PE(18:1_18:2)->PS(18:1_18:2)**	3.462	PTDSS2
**PE(O-16:1/22:6)->PC(O-16:1/22:6)**	3.446	PEMT
**PE(16:0_20:2)->PC(16:0_20:2)**	3.427	PEMT
**PE(16:0_20:1)->PS(16:0_20:1)**	3.421	PTDSS2
**PE(O-16:1/16:1)->PC(O-16:1/16:1)**	3.41	PEMT
**PE(18:1_18:1)->PS(18:1_18:1)**	3.407	PTDSS2
**DG(16:1_20:3)->PC(16:1_20:3)**	3.316	CHPT1
**PE(16:1_18:1)->PC(16:1_18:1)->PA(16:1_18:1)->PI(16:1_18:1)**	3.299	PEMT, PLD1, PLD2, CDS1, CDS2, CDIPT
**PA(18:0_20:5)->PI(18:0_20:5)**	3.245	CDS1, CDS2, CDIPT
**PG(34:0)->LPG(18:0)**	3.229	PLA2G4D, PLA2G4F, CRLS1
**PE(17:0_20:4)->PC(17:0_20:4)**	3.221	PEMT
**PE(O-17:0/20:5)->PC(O-17:0/20:5)**	3.217	PEMT
**PE(18:1_22:5)->PC(18:1_22:5)**	3.214	PEMT
**Cer(36:2->#8594->O2)**	3.184	SGMS1, SGMS2, CERT1

**PE(16:0_16:0)->PC(16:0_16:0)**	3.16	PEMT
**PE(18:0_22:6)->PC(18:0_22:6)**	3.154	PEMT
**PA(18:1_18:2)->PS(18:1_18:2)**	3.152	CDS1, PTDSS1
**PC(18:1_18:2)->PS(18:1_18:2)**	3.145	PTDSS1
**PE(18:1_20:3)->PS(18:1_20:3)**	3.118	PTDSS2
**PI(34:0)->LPI(18:0)->PI(40:6)**	3.076	PLA2G4D, PLA2G4E, MBOAT7
**PA(18:1_20:4)->PG(18:1_20:4)**	3.052	CDS1, CDS2, PTPMT1
**PE(16:0_18:0)->PS(16:0_18:0)**	3.022	PTDSS2
**PG(36:4)->LPG(20:4)**	2.984	PLA2G4D, PLA2G4F, CRLS1
**PE(18:0_18:3)->PC(18:0_18:3)**	2.979	PEMT
**PE(O-16:1/16:0)->PC(O-16:1/16:0)**	2.964	PEMT
**DG(18:1_22:5)->PC(18:1_22:5)**	2.96	CHPT1
**Cer(42:2->#8594->O2)**	2.945	SGMS1, SGMS2, CERT1
**PC(16:0_18:2)->PS(16:0_18:2)**	2.934	PTDSS1
**PE(O-16:1/20:5)->PC(O-16:1/20:5)**	2.894	PEMT
**PC(18:1_20:3)->PS(18:1_20:3)**	2.858	PTDSS1
**LPC(16:1)->PC(32:2)**	2.83	LPCAT1, LPCAT2, LPCAT3, LPCAT4
**PE(O-17:0/17:1)->PC(O-17:0/17:1)**	2.829	PEMT
**PE(O-16:1/18:1)->PC(O-16:1/18:1)**	2.801	PEMT
**DG(18:1_20:1)->PC(18:1_20:1)->PS(18:1_20:1)**	2.799	CHPT1, PTDSS1
**PS(34:1)->LPS(18:1)**	2.697	PLA2G2E, PLA2G2A, PLA2G2F
**DG(16:0_22:6)->PC(16:0_22:6)**	2.689	CHPT1
**PA(16:0_18:2)->PS(16:0_18:2)**	2.644	CDS1, PTDSS1
**DG(18:1_22:6)->PE(18:1_22:6)**	2.619	CEPT1
**LPI(18:1)->PI(38:5)**	2.614	MBOAT7
**PC(34:1)->LPC(18:0)**	2.582	PLA2G2E, PLA2G2A, PLA2G2D, PLA2G2F, PLA2G1B, PLA2G4A, PLA2G4B, PLA2G4C, PLA2G4D, PLA2G4E, PLA2G4F
**PE(16:1_18:0)->PC(16:1_18:0)**	2.542	PEMT
**PE(18:2_20:1)->PS(18:2_20:1)**	2.513	PTDSS2

**PE(20:1_20:4)->PC(20:1_20:4)->PS(20:1_20:4)**	2.512	PEMT, PTDSS1
**PC(32:0)->LPC(16:0)**	2.423	PLA2G2E, PLA2G2A, PLA2G2D, PLA2G2F, PLA2G1B, PLA2G4A, PLA2G4B, PLA2G4C, PLA2G4D, PLA2G4E, PLA2G4F
**DG(18:0_22:6)->PC(18:0_22:6)**	2.416	CHPT1
**PE(O-18:1/22:4)->PC(O-18:1/22:4)**	2.413	PEMT
**Cer(34:2->#8594->O2)**	2.404	SGMS1, SGMS2, CERT1
**PE(34:1)->LPE(18:1)**	2.403	PLA2G4C
**PI(36:3)->LPI(20:3)**	2.392	PLA2G4D, PLA2G4E
**PE(16:0_18:1)->PC(16:0_18:1)->PA(16:0_18:1)**	2.382	PEMT, PLD1, PLD2
**PE(O-16:0/20:5)->PC(O-16:0/20:5)**	2.356	PEMT
**LPE(20:4)->PE(36:5)**	2.309	MBOAT1, MBOAT2, LPCAT3, LPCAT4
**PE(34:0)->LPE(18:0)**	2.287	PLA2G4C
**DG(16:1_20:4)->PE(16:1_20:4)**	2.279	CEPT1
**PE(18:0_18:1)->PC(18:0_18:1)**	2.266	PEMT
**PE(18:0_22:5)->PC(18:0_22:5)->DG(18:0_22:5)**	2.174	PEMT
**PE(O-18:0/20:5)->PC(O-18:0/20:5)**	2.174	PEMT
**PE(O-16:2/18:1)->PC(O-16:2/18:1)**	2.14	PEMT
**PE(18:1_18:3)->PC(18:1_18:3)->DG(18:1_18:3)**	2.123	PEMT
**DG(18:0_20:2)->PC(18:0_20:2)**	2.12	CHPT1
**PG(34:2)->LPG(18:2)**	2.118	PLA2G4D, PLA2G4F, CRLS1
**PE(O-16:2/16:0)->PC(O-16:2/16:0)**	2.093	PEMT
**PE(O-18:1/18:1)->PC(O-18:1/18:1)**	2.054	PEMT
**PE(32:0)->LPE(16:0)**	1.993	PLA2G4C
**PE(18:0_18:2)->PC(18:0_18:2)**	1.99	PEMT
**PA(16:1_18:1)->PI(16:1_18:1)**	1.987	CDS1, CDS2, CDIPT
**PE(18:0_20:2)->PS(18:0_20:2)**	1.97	PTDSS2
**PI(34:2)->LPI(18:2)->PI(34:3)**	1.963	PLA2G4D, PLA2G4E, MBOAT7

**PC(18:1_20:1)->PS(18:1_20:1)**	1.916	PTDSS1
**DG(18:0_18:1)->PC(18:0_18:1)**	1.906	CHPT1
**PE(17:0_18:2)->PC(17:0_18:2)**	1.896	PEMT
**TG(50:1)->DG(34:1)**	1.893	PNPLA4, PNPLA5
**PE(O-16:1/22:5)->PC(O-16:1/22:5)**	1.882	PEMT
**PE(18:0_22:4)->PC(18:0_22:4)->PS(18:0_22:4)**	1.864	PEMT, PTDSS1
**DG(18:2_22:5)->PC(18:2_22:5)**	1.833	CHPT1
**PE(O-17:1/18:2)->PC(O-17:1/18:2)**	1.825	PEMT
**PE(O-18:2/22:6)->PC(O-18:2/22:6)**	1.818	PEMT
**Cer(36:1->#8594->O2)**	1.818	SGMS1, SGMS2, CERT1
**PE(O-18:1/20:4)->PC(O-18:1/20:4)**	1.816	PEMT
**DG(16:0_18:1)->PC(16:0_18:1)**	1.788	CHPT1
**PE(16:0_16:1)->PC(16:0_16:1)->DG(16:0_16:1)**	1.778	PEMT
**PE(O-16:0/18:2)->PC(O-16:0/18:2)**	1.749	PEMT
**PE(16:0_20:4)->PC(16:0_20:4)->PS(16:0_20:4)**	1.742	PEMT, PTDSS1
**TG(48:0)->DG(32:0)**	1.741	PNPLA4, PNPLA5
**PE(18:0_20:4)->PC(18:0_20:4)->DG(18:0_20:4)**	1.686	PEMT
**PA(16:1_18:0)->PS(16:1_18:0)->PE(16:1_18:0)->PC(16:1_18:0)->DG(16:1_18:0)**	1.655	CDS1, PTDSS1, PISD, PEMT
**lipid species suppressed reaction (AIC vs. Control)**		
**Reactions chains**	Z-score	Predicted genes
**PC(14:0_22:5)->DG(14:0_22:5)**	-5.809	
**DG(36:5)->TG(58:5)**	-5.628	DGAT2
**PC(17:1_18:0)->DG(17:1_18:0)**	-4.895	
**LPC(16:0)->LPA(16:0)->PA(38:5)**	-4.735	ENPP2, AGPAT1, AGPAT2, AGPAT3, AGPAT4, AGPAT5

**PS(18:1_22:4)->PE(18:1_22:4)**	-4.648	PISD
**LPE(16:0)->PE(38:4)->LPE(22:4)**	-4.516	MBOAT1, MBOAT2, LPCAT3, LPCAT4, PLA2G4C
**LPC(16:0)->PC(38:6)->LPC(22:6)**	-4.494	LPCAT1, LPCAT2, LPCAT3, LPCAT4, PLA2G2E, PLA2G2A, PLA2G2D, PLA2G2F, PLA2G1B, PLA2G4A, PLA2G4B, PLA2G4C, PLA2G4D, PLA2G4E, PLA2G4F
**LPC(16:0)->LPA(16:0)->PA(34:2)**	-4.458	ENPP2, AGPAT1, AGPAT2, AGPAT3, AGPAT4, AGPAT5
**LPC(18:0)->PC(40:4)**	-4.388	LPCAT1, LPCAT2, LPCAT3, LPCAT4
**PS(18:1_20:1)->PE(18:1_20:1)**	-4.333	PISD
**LPC(18:0)->PC(38:3)**	-4.288	LPCAT1, LPCAT2, LPCAT3, LPCAT4
**LPC(16:0)->LPA(16:0)->PA(36:3)**	-4.239	ENPP2, AGPAT1, AGPAT2, AGPAT3, AGPAT4, AGPAT5
**PE(38:4)->LPE(22:4)**	-4.127	PLA2G4C
**LPC(18:0)->LPA(18:0)**	-4.066	ENPP2
**PC(18:0_20:5)->PA(18:0_20:5)**	-4.064	PLD1, PLD2
**PS(20:0_20:4)->PE(20:0_20:4)**	-4.023	PISD
**LPC(18:0)->PC(40:5)**	-3.921	LPCAT1, LPCAT2, LPCAT3, LPCAT4
**LPC(16:0)->PC(36:2)**	-3.897	LPCAT1, LPCAT2, LPCAT3, LPCAT4
**PS(20:1_20:4)->PE(20:1_20:4)**	-3.893	PISD
**PS(16:0_18:2)->PE(16:0_18:2)**	-3.885	PISD
**LPC(16:0)->PC(36:3)**	-3.88	LPCAT1, LPCAT2, LPCAT3, LPCAT4
**LPC(16:0)->PC(34:2)**	-3.874	LPCAT1, LPCAT2, LPCAT3, LPCAT4
**LPE(18:0)->PE(40:4)**	-3.855	MBOAT1, MBOAT2, LPCAT3, LPCAT4
**LPE(16:0)->PE(36:2)->LPE(20:2)**	-3.843	MBOAT1, MBOAT2, LPCAT3, LPCAT4, PLA2G4C
**LPA(16:0)->PA(34:0)**	-3.835	AGPAT1, AGPAT2, AGPAT3, AGPAT4, AGPAT5
**PC(38:6)->LPC(22:6)**	-3.799	PLA2G2E, PLA2G2A, PLA2G2D, PLA2G2F, PLA2G1B, PLA2G4A, PLA2G4B, PLA2G4C, PLA2G4D, PLA2G4E, PLA2G4F
**LPI(18:2)->PI(36:4)**	-3.788	MBOAT7
**PS(18:1_18:2)->PE(18:1_18:2)**	-3.761	PISD
**PE(O-16:0/20:4)->PC(O-16:0/20:4)**	-3.761	PEMT
**PS(16:0_18:0)->PE(16:0_18:0)**	-3.741	PISD
**DG(16:0_18:0)->PA(16:0_18:0)**	-3.726	DGKA, DGKB, DGKD, DGKE, DGKG, DGKH, DGKI, DGKK, DGKQ, DGKZ

**PS(16:0_20:1)->PE(16:0_20:1)->PC(16:0_20:1)->DG(16:0_20:1)**	-3.714	PISD, PEMT
**LPE(16:0)->PE(38:5)->LPE(22:5)**	-3.669	MBOAT1, MBOAT2, LPCAT3, LPCAT4, PLA2G4C
**PS(18:2_18:2)->PE(18:2_18:2)**	-3.652	PISD
**LPA(16:0)->PA(38:5)**	-3.651	AGPAT1, AGPAT2, AGPAT3, AGPAT4, AGPAT5
**LPC(16:0)->PC(38:4)**	-3.633	LPCAT1, LPCAT2, LPCAT3, LPCAT4
**LPC(18:0)->PC(38:2)**	-3.599	LPCAT1, LPCAT2, LPCAT3, LPCAT4
**LPC(16:0)->PC(32:0)**	-3.594	LPCAT1, LPCAT2, LPCAT3, LPCAT4
**PE(O-18:2/20:2)->PC(O-18:2/20:2)**	-3.582	PEMT
**LPS(20:4)->PS(36:4)**	-3.553	MBOAT1, LPCAT3, LPCAT4
**PS(18:1_18:1)->PE(18:1_18:1)**	-3.522	PISD
**PC(16:1_20:4)->DG(16:1_20:4)**	-3.504	
**LPC(16:0)->PC(38:5)**	-3.486	LPCAT1, LPCAT2, LPCAT3, LPCAT4
**PC(16:1_20:3)->DG(16:1_20:3)**	-3.465	
**LPE(18:0)->PE(38:1)**	-3.457	MBOAT1, MBOAT2, LPCAT3, LPCAT4
**LPC(16:0)->PC(32:1)->LPC(16:1)->PC(36:5)->LPC(20:4)**	-3.426	LPCAT1, LPCAT2, LPCAT3, LPCAT4, PLA2G2E, PLA2G2A, PLA2G2D, PLA2G2F, PLA2G1B, PLA2G4A, PLA2G4B, PLA2G4C, PLA2G4D, PLA2G4E, PLA2G4F, LPCAT1, LPCAT2, LPCAT3, LPCAT4, PLA2G2E, PLA2G2A, PLA2G2D, PLA2G2F, PLA2G1B, PLA2G4A, PLA2G4B, PLA2G4C, PLA2G4D, PLA2G4E, PLA2G4F
**LPE(18:0)->PE(40:5)**	-3.403	MBOAT1, MBOAT2, LPCAT3, LPCAT4
**PS(18:0_22:4)->PE(18:0_22:4)**	-3.367	PISD
**PC(15:0_18:1)->DG(15:0_18:1)**	-3.357	
**PC(16:0_17:1)->DG(16:0_17:1)**	-3.339	
**PC(17:1_18:1)->DG(17:1_18:1)**	-3.335	
**LPC(16:0)->PC(36:1)**	-3.328	LPCAT1, LPCAT2, LPCAT3, LPCAT4
**PS(18:0_20:1)->PE(18:0_20:1)**	-3.301	PISD
**PE(O-16:0/22:4)->PC(O-16:0/22:4)**	-3.284	PEMT
**LPE(18:0)->PE(38:2)**	-3.282	MBOAT1, MBOAT2, LPCAT3, LPCAT4
**LPC(18:0)->PC(40:6)**	-3.279	LPCAT1, LPCAT2, LPCAT3, LPCAT4
**PC(18:1_22:5)->DG(18:1_22:5)**	-3.268	

**LPC(16:0)->LPA(16:0)->PA(36:2)**	-3.265	ENPP2, AGPAT1, AGPAT2, AGPAT3, AGPAT4, AGPAT5
**LPA(16:0)->PA(34:2)**	-3.259	AGPAT1, AGPAT2, AGPAT3, AGPAT4, AGPAT5
**PE(38:5)->LPE(22:5)**	-3.249	PLA2G4C
**LPC(16:0)->PC(36:4)**	-3.209	LPCAT1, LPCAT2, LPCAT3, LPCAT4
**LPE(16:0)->PE(34:2)**	-3.205	MBOAT1, MBOAT2, LPCAT3, LPCAT4
**LPS(18:1)->PS(40:5)**	-3.2	MBOAT1, LPCAT3, LPCAT4
**LPS(18:1)->PS(34:2)**	-3.165	MBOAT1, LPCAT3, LPCAT4
**DG(35:1)->TG(55:4)**	-3.084	DGAT2
**PE(O-16:1/20:3)->PC(O-16:1/20:3)**	-3.083	PEMT
**PC(18:0_18:1)->DG(18:0_18:1)**	-3.074	
**PE(16:0_22:4)->PC(16:0_22:4)->DG(16:0_22:4)**	-3.058	PEMT
**PC(18:1_18:3)->DG(18:1_18:3)->PE(18:1_18:3)**	-3.041	CEPT1
**LPI(18:0)->PI(34:0)**	-3.017	MBOAT7
**SM(36:2->#8594->O2)**	-3.007	SMPD1, SMPD4
**LPC(16:0)->PC(34:1)->LPC(18:1)->PC(40:7)**	-3.007	LPCAT1, LPCAT2, LPCAT3, LPCAT4, PLA2G2E, PLA2G2A, PLA2G2D, PLA2G2F, PLA2G1B, PLA2G4A, PLA2G4B, PLA2G4C, PLA2G4D, PLA2G4E, PLA2G4F, LPCAT1, LPCAT2, LPCAT3, LPCAT4
**PE(O-16:0/20:3)->PC(O-16:0/20:3)**	-2.999	PEMT
**DG(17:0_18:2)->PE(17:0_18:2)**	-2.992	CEPT1
**LPG(16:0)->PG(38:4)**	-2.991	LPGAT1, CRLS1
**LPG(16:0)->PG(34:2)**	-2.989	LPGAT1, CRLS1
**PC(16:0_18:1)->DG(16:0_18:1)->PA(16:0_18:1)->PI(16:0_18:1)**	-2.976	DGKA, DGKB, DGKD, DGKE, DGKG, DGKH, DGKI, DGKK, DGKQ, DGKZ, CDS1, CDS2, CDIPT
**PS(16:0_20:3)->PE(16:0_20:3)**	-2.968	PISD
**LPA(16:0)->PA(36:3)**	-2.95	AGPAT1, AGPAT2, AGPAT3, AGPAT4, AGPAT5
**PS(38:0)->LPS(22:0)->PS(38:1)**	-2.93	PLA2G2E, PLA2G2A, PLA2G2F, MBOAT1, LPCAT3, LPCAT4
**SM(42:2->#8594->O2)**	-2.923	SMPD1, SMPD4
**LPG(16:0)->PG(34:0)**	-2.896	LPGAT1, CRLS1
**LPG(16:0)->PG(36:3)**	-2.872	LPGAT1, CRLS1

**LPS(18:1)->PS(36:1)**	-2.86	MBOAT1, LPCAT3, LPCAT4
**PE(O-18:1/18:3)->PC(O-18:1/18:3)**	-2.858	PEMT
**LPS(18:1)->PS(38:5)**	-2.836	MBOAT1, LPCAT3, LPCAT4
**LPE(16:0)->PE(36:3)**	-2.825	MBOAT1, MBOAT2, LPCAT3, LPCAT4
**PC(16:0_18:1)->DG(16:0_18:1)->PE(16:0_18:1)**	-2.822	CEPT1
**LPS(18:1)->PS(38:4)**	-2.816	MBOAT1, LPCAT3, LPCAT4
**LPE(18:0)->PE(38:3)**	-2.795	MBOAT1, MBOAT2, LPCAT3, LPCAT4
**PC(18:1_18:1)->DG(18:1_18:1)->PA(18:1_18:1)->PI(18:1_18:1)**	-2.771	DGKA, DGKB, DGKD, DGKE, DGKG, DGKH, DGKI, DGKK, DGKQ, DGKZ, CDS1, CDS2, CDIPT
**LPE(16:0)->PE(32:1)**	-2.752	MBOAT1, MBOAT2, LPCAT3, LPCAT4
**PC(32:1)->LPC(16:1)**	-2.748	PLA2G2E, PLA2G2A, PLA2G2D, PLA2G2F, PLA2G1B, PLA2G4A, PLA2G4B, PLA2G4C, PLA2G4D, PLA2G4E, PLA2G4F
**PS(18:1_20:3)->PE(18:1_20:3)**	-2.699	PISD
**PC(16:1_18:1)->DG(16:1_18:1)->PA(16:1_18:1)->PG(16:1_18:1)**	-2.681	DGKA, DGKB, DGKD, DGKE, DGKG, DGKH, DGKI, DGKK, DGKQ, DGKZ, CDS1, CDS2, PTPMT1
**PE(O-18:2/16:0)->PC(O-18:2/16:0)**	-2.679	PEMT
**PE(36:2)->LPE(20:2)**	-2.658	PLA2G4C
**PC(16:0_18:1)->DG(16:0_18:1)->PA(16:0_18:1)->PS(16:0_18:1)**	-2.647	DGKA, DGKB, DGKD, DGKE, DGKG, DGKH, DGKI, DGKK, DGKQ, DGKZ, CDS1, PTDSS1
**PS(18:2_20:1)->PE(18:2_20:1)**	-2.638	PISD
**LPS(18:1)->PS(38:3)**	-2.631	MBOAT1, LPCAT3, LPCAT4
**PC(16:0_22:6)->DG(16:0_22:6)**	-2.63	
**PC(18:0_20:2)->DG(18:0_20:2)**	-2.623	
**PC(16:0_18:1)->DG(16:0_18:1)->PA(16:0_18:1)->PG(16:0_18:1)**	-2.592	DGKA, DGKB, DGKD, DGKE, DGKG, DGKH, DGKI, DGKK, DGKQ, DGKZ, CDS1, CDS2, PTPMT1
**LPE(16:0)->PE(34:0)**	-2.579	MBOAT1, MBOAT2, LPCAT3, LPCAT4
**LPG(16:0)->PG(36:1)**	-2.573	LPGAT1, CRLS1
**LPS(18:1)->PS(40:6)->LPS(22:6)**	-2.572	MBOAT1, LPCAT3, LPCAT4, PLA2G2E, PLA2G2A, PLA2G2F
**LPS(18:1)->PS(34:1)**	-2.571	MBOAT1, LPCAT3, LPCAT4
**DG(18:0_22:5)->PE(18:0_22:5)**	-2.571	CEPT1
**PS(18:0_20:2)->PE(18:0_20:2)**	-2.558	PISD

**DG(36:1)->TG(58:7)**	-2.551	DGAT2
**PS(38:0)->LPS(22:0)->PS(40:2)**	-2.541	PLA2G2E, PLA2G2A, PLA2G2F, MBOAT1, LPCAT3, LPCAT4
**LPG(16:0)->PG(38:6)**	-2.533	LPGAT1, CRLS1
**PC(18:0_22:5)->PS(18:0_22:5)->PE(18:0_22:5)**	-2.519	PTDSS1, PISD
**LPI(18:0)->PI(36:1)**	-2.519	MBOAT7
**PC(18:2_18:2)->DG(18:2_18:2)->PE(18:2_18:2)**	-2.517	CEPT1
**LPE(16:0)->PE(32:0)**	-2.512	MBOAT1, MBOAT2, LPCAT3, LPCAT4
**PA(18:1_18:1)->DG(18:1_18:1)->PE(18:1_18:1)**	-2.5	PLPP1, PLPP2, PLPP3, CEPT1
**LPI(18:0)->PI(34:1)->LPI(18:1)->PI(34:2)**	-2.498	MBOAT7, PLA2G4D, PLA2G4E, MBOAT7
**LPG(16:0)->PG(36:4)**	-2.49	LPGAT1, CRLS1
**DG(30:0)->TG(52:6)**	-2.479	DGAT2
**PC(14:0_18:1)->DG(14:0_18:1)->PE(14:0_18:1)**	-2.479	CEPT1
**LPI(18:0)->PI(36:2)**	-2.464	MBOAT7
**LPI(18:0)->PI(34:1)->LPI(18:1)->PI(36:3)**	-2.448	MBOAT7, PLA2G4D, PLA2G4E, MBOAT7
**PC(18:2_22:5)->DG(18:2_22:5)**	-2.44	
**LPS(18:1)->PS(36:2)**	-2.435	MBOAT1, LPCAT3, LPCAT4
**LPE(16:0)->PE(36:1)->LPE(20:1)**	-2.433	MBOAT1, MBOAT2, LPCAT3, LPCAT4, PLA2G4C
**PE(O-18:0/20:4)->PC(O-18:0/20:4)**	-2.411	PEMT
**PS(18:1_20:0)->PE(18:1_20:0)**	-2.383	PISD
**LPE(16:0)->PE(34:1)**	-2.373	MBOAT1, MBOAT2, LPCAT3, LPCAT4
**PA(18:1_18:1)->PI(18:1_18:1)**	-2.368	CDS1, CDS2, CDIPT
**DG(36:1)->TG(58:6)**	-2.364	DGAT2
**DG(30:0)->TG(48:0)**	-2.359	DGAT2
**SM(34:2->#8594->O2)**	-2.332	SMPD1, SMPD4
**DG(30:1)->TG(52:1)**	-2.326	DGAT2
**LPC(16:0)->LPA(16:0)->PA(34:1)->LPA(18:1)**	-2.251	ENPP2, AGPAT1, AGPAT2, AGPAT3, AGPAT4, AGPAT5, PLA2G4A, PLA2G4D, PLA2G4E, PLA2G4F

**PC(34:3)->LPC(18:2)**	-2.243	PLA2G2E, PLA2G2A, PLA2G2D, PLA2G2F, PLA2G1B, PLA2G4A, PLA2G4B, PLA2G4C, PLA2G4D, PLA2G4E, PLA2G4F
**PC(36:5)->LPC(20:4)**	-2.225	PLA2G2E, PLA2G2A, PLA2G2D, PLA2G2F, PLA2G1B, PLA2G4A, PLA2G4B, PLA2G4C, PLA2G4D, PLA2G4E, PLA2G4F
**PC(16:0_22:4)->DG(16:0_22:4)**	-2.214	
**DG(16:0_16:1)->PE(16:0_16:1)**	-2.209	CEPT1
**PC(18:1_22:4)->DG(18:1_22:4)->PE(18:1_22:4)**	-2.193	CEPT1
**LPE(16:0)->PE(38:6)->LPE(22:6)**	-2.189	MBOAT1, MBOAT2, LPCAT3, LPCAT4, PLA2G4C
**PS(16:0_18:1)->PE(16:0_18:1)**	-2.187	PISD
**SM(40:2->#8594->O2)**	-2.164	SMPD1, SMPD4
**PE(O-18:2/18:0)->PC(O-18:2/18:0)**	-2.146	PEMT
**PC(18:1_18:1)->DG(18:1_18:1)->PA(18:1_18:1)->PS(18:1_18:1)**	-2.138	DGKA, DGKB, DGKD, DGKE, DGKG, DGKH, DGKI, DGKK, DGKQ, DGKZ, CDS1, PTDSS1
**PC(16:1_18:1)->DG(16:1_18:1)->PE(16:1_18:1)**	-2.136	CEPT1
**PC(18:1_20:1)->DG(18:1_20:1)->PE(18:1_20:1)**	-2.122	CEPT1
**PC(18:2_20:2)->DG(18:2_20:2)**	-2.12	
**LPE(16:0)->PE(36:4)->LPE(20:4)**	-2.119	MBOAT1, MBOAT2, LPCAT3, LPCAT4, PLA2G4C
**LPS(18:1)->PS(38:2)**	-2.118	MBOAT1, LPCAT3, LPCAT4
**PC(18:1_18:1)->DG(18:1_18:1)->PA(18:1_18:1)->PG(18:1_18:1)**	-2.109	DGKA, DGKB, DGKD, DGKE, DGKG, DGKH, DGKI, DGKK, DGKQ, DGKZ, CDS1, CDS2, PTPMT1
**DG(32:1)->TG(54:7)**	-2.102	DGAT2
**PC(18:1_22:6)->DG(18:1_22:6)**	-2.087	
**DG(35:1)->TG(55:3)**	-2.084	DGAT2
**PS(16:1_22:4)->PE(16:1_22:4)**	-2.073	PISD
**LPC(16:0)->PC(32:1)->LPC(16:1)->PC(34:3)->LPC(18:2)->LPA(18:2)**	-2.073	LPCAT1, LPCAT2, LPCAT3, LPCAT4, PLA2G2E, PLA2G2A, PLA2G2D, PLA2G2F, PLA2G1B, PLA2G4A, PLA2G4B, PLA2G4C, PLA2G4D, PLA2G4E, PLA2G4F, LPCAT1, LPCAT2, LPCAT3, LPCAT4, PLA2G2E, PLA2G2A, PLA2G2D, PLA2G2F, PLA2G1B, PLA2G4A, PLA2G4B, PLA2G4C, PLA2G4D, PLA2G4E, PLA2G4F, ENPP2
**PC(18:1_18:1)->DG(18:1_18:1)->PE(18:1_18:1)**	-2.069	CEPT1

**PA(18:1_18:1)->DG(18:1_18:1)->PC(18:1_18:1)**	-2.06	PLPP1, PLPP2, PLPP3, CHPT1
**SM(36:1->#8594->O2)**	-2.046	SMPD1, SMPD4
**PS(38:0)->LPS(22:0)->PS(42:3)**	-2.017	PLA2G2E, PLA2G2A, PLA2G2F, MBOAT1, LPCAT3, LPCAT4
**PC(16:0_20:1)->DG(16:0_20:1)**	-2	
**PA(16:0_18:1)->DG(16:0_18:1)->PE(16:0_18:1)**	-1.999	PLPP1, PLPP2, PLPP3, CEPT1
**PC(16:1_18:2)->DG(16:1_18:2)**	-1.985	
**PC(16:0_20:3)->DG(16:0_20:3)**	-1.973	
**PC(16:1_18:0)->PA(16:1_18:0)->DG(16:1_18:0)->PE(16:1_18:0)**	-1.954	PLD1, PLD2, PLPP1, PLPP2, PLPP3, CEPT1
**DG(33:1)->TG(53:4)**	-1.953	DGAT2
**LPC(16:0)->PC(32:1)->LPC(16:1)->PC(32:2)**	-1.95	LPCAT1, LPCAT2, LPCAT3, LPCAT4, PLA2G2E, PLA2G2A, PLA2G2D, PLA2G2F, PLA2G1B, PLA2G4A, PLA2G4B, PLA2G4C, PLA2G4D, PLA2G4E, PLA2G4F, LPCAT1, LPCAT2, LPCAT3, LPCAT4
**PC(16:0_16:0)->DG(16:0_16:0)->PE(16:0_16:0)**	-1.947	CEPT1
**PC(14:0_16:0)->DG(14:0_16:0)**	-1.932	
**PC(16:0_22:5)->DG(16:0_22:5)**	-1.931	
**PC(18:0_18:3)->DG(18:0_18:3)->PE(18:0_18:3)**	-1.928	CEPT1
**PE(O-17:1/22:6)->PC(O-17:1/22:6)**	-1.916	PEMT
**PC(18:0_18:2)->DG(18:0_18:2)->PA(18:0_18:2)**	-1.888	DGKA, DGKB, DGKD, DGKE, DGKG, DGKH, DGKI, DGKK, DGKQ, DGKZ
**PA(16:1_18:1)->PG(16:1_18:1)**	-1.878	CDS1, CDS2, PTPMT1
**DG(18:1_18:3)->PE(18:1_18:3)**	-1.878	CEPT1
**PC(18:2_20:4)->DG(18:2_20:4)**	-1.875	
**LPI(18:0)->PI(38:3)**	-1.863	MBOAT7
**DG(30:0)->TG(52:4)**	-1.856	DGAT2
**PC(18:1_20:3)->DG(18:1_20:3)->PE(18:1_20:3)**	-1.843	CEPT1
**LPC(16:0)->LPA(16:0)->PA(34:0)->LPA(18:0)**	-1.838	ENPP2, AGPAT1, AGPAT2, AGPAT3, AGPAT4, AGPAT5, PLA2G4A, PLA2G4D, PLA2G4E, PLA2G4F

**PC(18:0_22:6)->PS(18:0_22:6)**	-1.835	PTDSS1
**PC(16:0_20:4)->DG(16:0_20:4)->PE(16:0_20:4)**	-1.828	CEPT1
**DG(36:4)->TG(58:8)**	-1.815	DGAT2
**PC(14:1_18:1)->DG(14:1_18:1)**	-1.81	
**PS(16:0_20:4)->PE(16:0_20:4)**	-1.806	PISD
**LPG(16:0)->PG(36:2)**	-1.797	LPGAT1, CRLS1
**PC(16:1_18:0)->PA(16:1_18:0)->PI(16:1_18:0)**	-1.793	PLD1, PLD2, CDS1, CDS2, CDIPT
**PS(40:6)->LPS(22:6)**	-1.787	PLA2G2E, PLA2G2A, PLA2G2F
**PE(18:0_20:5)->PC(18:0_20:5)->PA(18:0_20:5)->PI(18:0_20:5)**	-1.782	PEMT, PLD1, PLD2, CDS1, CDS2, CDIPT
**LPA(16:0)->PA(34:1)**	-1.777	AGPAT1, AGPAT2, AGPAT3, AGPAT4, AGPAT5
**LPG(16:0)->PG(38:5)**	-1.777	LPGAT1, CRLS1
**PC(18:0_18:2)->DG(18:0_18:2)->PE(18:0_18:2)**	-1.744	CEPT1
**PC(18:0_22:6)->DG(18:0_22:6)->PE(18:0_22:6)**	-1.744	CEPT1
**PC(18:1_20:4)->PA(18:1_20:4)->DG(18:1_20:4)**	-1.736	PLD1, PLD2, PLPP1, PLPP2, PLPP3
**PC(16:1_18:0)->DG(16:1_18:0)**	-1.733	
**LPC(18:1)->PC(40:7)**	-1.72	LPCAT1, LPCAT2, LPCAT3, LPCAT4
**PC(18:1_20:4)->DG(18:1_20:4)**	-1.718	
**PE(17:0_22:4)->PS(17:0_22:4)**	-1.702	PTDSS2
**LPE(18:0)->PE(40:6)**	-1.685	MBOAT1, MBOAT2, LPCAT3, LPCAT4
**PE(O-18:0/16:1)->PC(O-18:0/16:1)**	-1.676	PEMT
**PC(18:2_20:3)->DG(18:2_20:3)**	-1.676	
**PC(16:0_20:2)->DG(16:0_20:2)->PE(16:0_20:2)**	-1.66	CEPT1
**lipid species most suppressed reaction (AIC vs. Control)**		
**Reactions chains**		Predicted genes
**PC(14:0_22:5)->DG(14:0_22:5)**	-5.809	

**DG(36:5)->TG(58:5)**	-5.628	DGAT2
**PC(17:1_18:0)->DG(17:1_18:0)**	-4.895	
**PS(18:1_22:4)->PE(18:1_22:4)**	-4.648	PISD
**LPC(18:0)->PC(40:4)**	-4.388	LPCAT1, LPCAT2, LPCAT3, LPCAT4
**PS(18:1_20:1)->PE(18:1_20:1)**	-4.333	PISD
**PE(38:4)->LPE(22:4)**	-4.127	PLA2G4C
**PC(18:0_20:5)->PA(18:0_20:5)**	-4.064	PLD1, PLD2
**PS(20:0_20:4)->PE(20:0_20:4)**	-4.023	PISD
**LPC(16:0)->PC(36:2)**	-3.897	LPCAT1, LPCAT2, LPCAT3, LPCAT4
**PS(20:1_20:4)->PE(20:1_20:4)**	-3.893	PISD
**PS(16:0_18:2)->PE(16:0_18:2)**	-3.885	PISD
**LPE(18:0)->PE(40:4)**	-3.855	MBOAT1, MBOAT2, LPCAT3, LPCAT4
**LPA(16:0)->PA(34:0)**	-3.835	AGPAT1, AGPAT2, AGPAT3, AGPAT4, AGPAT5
**PC(38:6)->LPC(22:6)**	-3.799	PLA2G2E, PLA2G2A, PLA2G2D, PLA2G2F, PLA2G1B, PLA2G4A, PLA2G4B, PLA2G4C, PLA2G4D, PLA2G4E, PLA2G4F
**LPI(18:2)->PI(36:4)**	-3.788	MBOAT7
**PS(18:1_18:2)->PE(18:1_18:2)**	-3.761	PISD
**PE(O-16:0/20:4)->PC(O-16:0/20:4)**	-3.761	PEMT
**PS(16:0_18:0)->PE(16:0_18:0)**	-3.741	PISD
**DG(16:0_18:0)->PA(16:0_18:0)**	-3.726	DGKA, DGKB, DGKD, DGKE, DGKG, DGKH, DGKI, DGKK, DGKQ, DGKZ
**PS(16:0_20:1)->PE(16:0_20:1)->PC(16:0_20:1)->DG(16:0_20:1)**	-3.714	PISD, PEMT
**PS(18:2_18:2)->PE(18:2_18:2)**	-3.652	PISD
**PE(O-18:2/20:2)->PC(O-18:2/20:2)**	-3.582	PEMT
**LPS(20:4)->PS(36:4)**	-3.553	MBOAT1, LPCAT3, LPCAT4
**PS(18:1_18:1)->PE(18:1_18:1)**	-3.522	PISD
**PC(16:1_20:4)->DG(16:1_20:4)**	-3.504	
**PC(16:1_20:3)->DG(16:1_20:3)**	-3.465	
**PS(18:0_22:4)->PE(18:0_22:4)**	-3.367	PISD

**PC(15:0_18:1)->DG(15:0_18:1)**	-3.357	
**PC(16:0_17:1)->DG(16:0_17:1)**	-3.339	
**PC(17:1_18:1)->DG(17:1_18:1)**	-3.335	
**PS(18:0_20:1)->PE(18:0_20:1)**	-3.301	PISD
**PE(O-16:0/22:4)->PC(O-16:0/22:4)**	-3.284	PEMT
**PC(18:1_22:5)->DG(18:1_22:5)**	-3.268	
**PE(38:5)->LPE(22:5)**	-3.249	PLA2G4C
**LPE(16:0)->PE(34:2)**	-3.205	MBOAT1, MBOAT2, LPCAT3, LPCAT4
**LPS(18:1)->PS(40:5)**	-3.2	MBOAT1, LPCAT3, LPCAT4
**DG(35:1)->TG(55:4)**	-3.084	DGAT2
**PE(O-16:1/20:3)->PC(O-16:1/20:3)**	-3.083	PEMT
**PC(18:0_18:1)->DG(18:0_18:1)**	-3.074	
**PE(16:0_22:4)->PC(16:0_22:4)->DG(16:0_22:4)**	-3.058	PEMT
**PC(18:1_18:3)->DG(18:1_18:3)->PE(18:1_18:3)**	-3.041	CEPT1
**LPI(18:0)->PI(34:0)**	-3.017	MBOAT7
**SM(36:2->#8594->O2)**	-3.007	SMPD1, SMPD4
**PE(O-16:0/20:3)->PC(O-16:0/20:3)**	-2.999	PEMT
**DG(17:0_18:2)->PE(17:0_18:2)**	-2.992	CEPT1
**LPG(16:0)->PG(38:4)**	-2.991	LPGAT1, CRLS1
**PS(16:0_20:3)->PE(16:0_20:3)**	-2.968	PISD
**PS(38:0)->LPS(22:0)->PS(38:1)**	-2.93	PLA2G2E, PLA2G2A, PLA2G2F, MBOAT1, LPCAT3, LPCAT4
**SM(42:2->#8594->O2)**	-2.923	SMPD1, SMPD4
**PE(O-18:1/18:3)->PC(O-18:1/18:3)**	-2.858	PEMT
**PC(16:0_18:1)->DG(16:0_18:1)->PE(16:0_18:1)**	-2.822	CEPT1
**PC(32:1)->LPC(16:1)**	-2.748	PLA2G2E, PLA2G2A, PLA2G2D, PLA2G2F, PLA2G1B, PLA2G4A, PLA2G4B, PLA2G4C, PLA2G4D, PLA2G4E, PLA2G4F
**PS(18:1_20:3)->PE(18:1_20:3)**	-2.699	PISD
**PE(O-18:2/16:0)->PC(O-18:2/16:0)**	-2.679	PEMT

**PE(36:2)->LPE(20:2)**	-2.658	PLA2G4C
**PS(18:2_20:1)->PE(18:2_20:1)**	-2.638	PISD
**PC(16:0_22:6)->DG(16:0_22:6)**	-2.63	
**PC(18:0_20:2)->DG(18:0_20:2)**	-2.623	
**DG(18:0_22:5)->PE(18:0_22:5)**	-2.571	CEPT1
**PS(18:0_20:2)->PE(18:0_20:2)**	-2.558	PISD
**DG(36:1)->TG(58:7)**	-2.551	DGAT2
**PC(18:0_22:5)->PS(18:0_22:5)->PE(18:0_22:5)**	-2.519	PTDSS1, PISD
**PC(18:2_18:2)->DG(18:2_18:2)->PE(18:2_18:2)**	-2.517	CEPT1
**PA(18:1_18:1)->DG(18:1_18:1)->PE(18:1_18:1)**	-2.5	PLPP1, PLPP2, PLPP3, CEPT1
**DG(30:0)->TG(52:6)**	-2.479	DGAT2
**PC(14:0_18:1)->DG(14:0_18:1)->PE(14:0_18:1)**	-2.479	CEPT1
**PC(18:2_22:5)->DG(18:2_22:5)**	-2.44	
**PE(O-18:0/20:4)->PC(O-18:0/20:4)**	-2.411	PEMT
**PS(18:1_20:0)->PE(18:1_20:0)**	-2.383	PISD
**SM(34:2->#8594->O2)**	-2.332	SMPD1, SMPD4
**DG(30:1)->TG(52:1)**	-2.326	DGAT2
**PC(34:3)->LPC(18:2)**	-2.243	PLA2G2E, PLA2G2A, PLA2G2D, PLA2G2F, PLA2G1B, PLA2G4A, PLA2G4B, PLA2G4C, PLA2G4D, PLA2G4E, PLA2G4F
**PC(36:5)->LPC(20:4)**	-2.225	PLA2G2E, PLA2G2A, PLA2G2D, PLA2G2F, PLA2G1B, PLA2G4A, PLA2G4B, PLA2G4C, PLA2G4D, PLA2G4E, PLA2G4F
**PC(16:0_22:4)->DG(16:0_22:4)**	-2.214	
**DG(16:0_16:1)->PE(16:0_16:1)**	-2.209	CEPT1
**PC(18:1_22:4)->DG(18:1_22:4)->PE(18:1_22:4)**	-2.193	CEPT1
**PS(16:0_18:1)->PE(16:0_18:1)**	-2.187	PISD
**SM(40:2->#8594->O2)**	-2.164	SMPD1, SMPD4
**PE(O-18:2/18:0)->PC(O-18:2/18:0)**	-2.146	PEMT

**PC(16:1_18:1)->DG(16:1_18:1)->PE(16:1_18:1)**	-2.136	CEPT1
**PC(18:1_20:1)->DG(18:1_20:1)->PE(18:1_20:1)**	-2.122	CEPT1
**PC(18:2_20:2)->DG(18:2_20:2)**	-2.12	
**DG(32:1)->TG(54:7)**	-2.102	DGAT2
**PC(18:1_22:6)->DG(18:1_22:6)**	-2.087	
**PS(16:1_22:4)->PE(16:1_22:4)**	-2.073	PISD
**PC(18:1_18:1)->DG(18:1_18:1)->PE(18:1_18:1)**	-2.069	CEPT1
**SM(36:1->#8594->O2)**	-2.046	SMPD1, SMPD4
**PC(16:0_20:1)->DG(16:0_20:1)**	-2	
**PA(16:0_18:1)->DG(16:0_18:1)->PE(16:0_18:1)**	-1.999	PLPP1, PLPP2, PLPP3, CEPT1
**PC(16:1_18:2)->DG(16:1_18:2)**	-1.985	
**PC(16:0_20:3)->DG(16:0_20:3)**	-1.973	
**PC(16:1_18:0)->PA(16:1_18:0)->DG(16:1_18:0)->PE(16:1_18:0)**	-1.954	PLD1, PLD2, PLPP1, PLPP2, PLPP3, CEPT1
**DG(33:1)->TG(53:4)**	-1.953	DGAT2
**PC(16:0_16:0)->DG(16:0_16:0)->PE(16:0_16:0)**	-1.947	CEPT1
**PC(14:0_16:0)->DG(14:0_16:0)**	-1.932	
**PC(16:0_22:5)->DG(16:0_22:5)**	-1.931	
**PC(18:0_18:3)->DG(18:0_18:3)->PE(18:0_18:3)**	-1.928	CEPT1
**PE(O-17:1/22:6)->PC(O-17:1/22:6)**	-1.916	PEMT
**PC(18:0_18:2)->DG(18:0_18:2)->PA(18:0_18:2)**	-1.888	DGKA, DGKB, DGKD, DGKE, DGKG, DGKH, DGKI, DGKK, DGKQ, DGKZ
**PA(16:1_18:1)->PG(16:1_18:1)**	-1.878	CDS1, CDS2, PTPMT1
**DG(18:1_18:3)->PE(18:1_18:3)**	-1.878	CEPT1
**PC(18:2_20:4)->DG(18:2_20:4)**	-1.875	
**PC(18:1_20:3)->DG(18:1_20:3)->PE(18:1_20:3)**	-1.843	CEPT1

**PC(16:0_20:4)->DG(16:0_20:4)->PE(16:0_20:4)**	-1.828	CEPT1
**DG(36:4)->TG(58:8)**	-1.815	DGAT2
**PC(14:1_18:1)->DG(14:1_18:1)**	-1.81	
**PS(16:0_20:4)->PE(16:0_20:4)**	-1.806	PISD
**PS(40:6)->LPS(22:6)**	-1.787	PLA2G2E, PLA2G2A, PLA2G2F
**PE(18:0_20:5)->PC(18:0_20:5)->PA(18:0_20:5)->PI(18:0_20:5)**	-1.782	PEMT, PLD1, PLD2, CDS1, CDS2, CDIPT
**PC(18:0_22:6)->DG(18:0_22:6)->PE(18:0_22:6)**	-1.744	CEPT1
**PC(18:1_20:4)->PA(18:1_20:4)->DG(18:1_20:4)**	-1.736	PLD1, PLD2, PLPP1, PLPP2, PLPP3
**LPC(18:1)->PC(40:7)**	-1.72	LPCAT1, LPCAT2, LPCAT3, LPCAT4
**PE(17:0_22:4)->PS(17:0_22:4)**	-1.702	PTDSS2
**PE(O-18:0/16:1)->PC(O-18:0/16:1)**	-1.676	PEMT
**PC(18:2_20:3)->DG(18:2_20:3)**	-1.676	
https://lipidmaps.org/data/proteome/LMPD_table.php?GENE_SYMBOL=CEPT2	https://lipidmaps.org/data/proteome/LMPD_table.php?GENE_SYMBOL=CEPT1	https://lipidmaps.org/data/proteome/LMPD_table.php?GENE_SYMBOL=CEPT0

Supplemental Table 3: Active and suppressed reactions of lipid classes and species using BioPAN by Lipid MAPS.
